# Evaluating Palladium 4d-to-2p X‑ray Emission Spectroscopy for Characterizing Catalytically Relevant Species

**DOI:** 10.1021/acs.inorgchem.5c04266

**Published:** 2026-01-09

**Authors:** Anna G. Scott, Sergey Peredkov, Angeles Lopez-Martin, Richard J. Lewis, Graham J. Hutchings, Serena DeBeer

**Affiliations:** † Max Planck Institute for Chemical Energy Conversion, D-45470 Mülheim an der Ruhr, Germany; ‡ Max Planck−Cardiff Centre on the Fundamentals of Heterogeneous Catalysis FUNCAT, Cardiff Catalysis Institute, School of Chemistry, 2112 Cardiff University, Cardiff CF24 4HQ, United Kingdom

## Abstract

X-ray absorption spectroscopy (XAS) has diverse applications in materials characterization and catalysis. While K-edge XAS can provide detailed information about electronic and geometric structures for 3d transition metals, its application to second- and third-row transition metals is often limited by substantial core–hole lifetime broadenings. For the later transition metals, higher d-electron counts further reduce the electronic structural information content due to weak and absent 1s-to-nd pre-edge features. L-shell X-ray emission spectroscopy (XES), specifically 4d-to-2p XES, can overcome these limitations by accessing transitions that are dipole allowed and have intrinsically narrower core–hole lifetime broadenings. Herein, the utility of Pd 4d-to-2p XES for in situ catalysis research in both homogeneous and heterogeneous systems is explored through the study of well-defined PdO_*x*_H_*y*_ complexes and Pd nanoparticles that are relevant to Pd-based catalysts.

## Graphical abstract

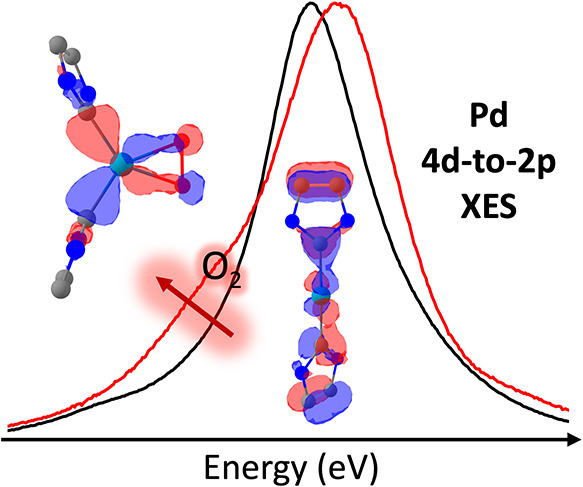

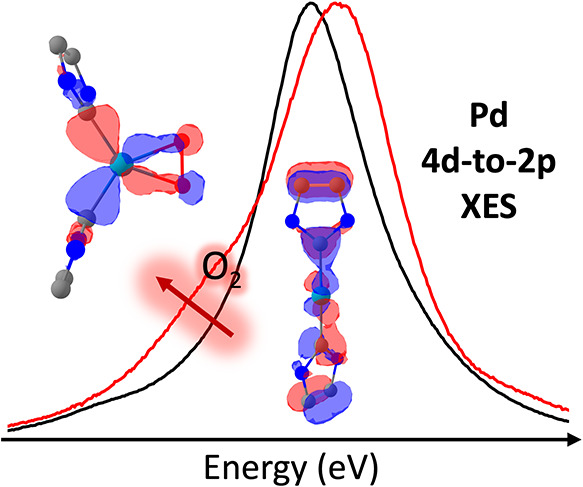

## Introduction

X-ray spectroscopy has diverse applications in molecular complex and materials characterization, including electronic structure investigations, in situ catalysis studies, and structure determination.^1–8^ It is an especially powerful technique for in situ catalyst characterization, as it is one of the few methods capable of characterizing catalysts under relevant conditions, including in the liquid phase, in the gas phase, and under high temperatures and pressures, and is element selective.^2,4–6,8,9^ However, only a limited number of X-ray spectroscopy techniques have been fully utilized and explored for identifying important catalytic intermediates, metal oxidation states, and dynamic catalyst structures towards evaluating and improving catalysts.^2,4,10^ One commonly used technique is K-edge X-ray absorption spectroscopy (XAS), which involves transitions from the 1s orbital to unfilled valence orbitals (pre-edge and XANES regions) and, at higher energies, to the continuum (EXAFS region).^2^ While the pre-edges of XAS spectra can provide rich information about the oxidation state, spin state, and coordination geometry for 3d transition metals, 4d and 5d transition metals suffer from broadening of features due to short 1s core–hole lifetimes.^2–4,6,9–11^ Additionally, metals with higher d-electron counts also have fewer available transitions to the valence d-orbitals, and thus, fewer transitions are observed to the unoccupied levels involved in ligand and reactant binding. As such, in situ K-edge XAS studies of later 4d transition metals are often limited in the electronic structural information that can be extracted.

X-ray emission spectroscopy (XES)-based methods have the potential to provide additional details complementary to traditional K-edge XAS studies because XES probes filled ligand and metal orbitals. In addition, spectral broadening in XES (as well as XAS) can be reduced by utilizing L-shell excitations, which have decreased core–hole lifetime broadenings. In XES, a core level electron is first ionized, and the resulting fluorescence is monitored as an electron fills the core–hole (Figure 1).^2^ By utilizing XES, the spectral resolution of K-edge XAS spectra can be improved by monitoring a single emission line as XAS energies are scanned, also known as high-energy resolution fluorescence-detected (HERFD) XAS.^2,9,10,14^ In HERFD XAS, the spectral resolution is increased if the final state core–hole lifetime broadening is less than that of the intermediate state.^2,9,10,14^ L-edge HERFD XAS spectra of Mo, Ru, Rh, Pd, and Pt compounds, for example, show increased resolution of the absorption edge, which can aid in the determination of metal oxidation state and coordination environment.^15–21^ Of particular interest for in situ studies of catalysts, however, is valence-to-core (VtC) XES. VtC XES probes specifically valence orbitals that are key for understanding catalyst mechanisms and providing insight for catalyst improvement (Figure 1). XES experiments can also be conducted using dispersive von Hámos spectrometers and are therefore readily amenable to time-resolved in situ catalysis studies.^2^ For third- and fourth-row elements, np-to-1s and ns-to-1s (K-shell) VtC XES has seen increased empirical and theoretical development^22,23^ in the past decade and has demonstrated the ability to identify ligands in metalloproteins,^24–26^ quantify small molecule bond activation and coordination geometry,^27–30^ determine metal oxidation and spin states,^31–33^ track biomolecule transformations,^34–36^ and elucidate catalyst structures.^37–42^ In 3d transition metal complexes, K-shell VtC XES is a detailed reporter of ligand identity, oxidation state, and binding mode.^2,33^ Transitions from ligand-based s- and p-orbitals to metal-based s-orbitals result in spectra with low-intensity features that gain intensity from increased metal–ligand covalency and overlap of metal–ligand orbitals.^12^ There has also been recent success in ligand VtC XES measurements that monitor transitions from ligand valence orbitals to ligand core s-orbitals, resulting in higher-intensity transitions.^42,43^ For 4d and 5d transition metals, studies of Nb and Mo K-shell VtC XES report the reduced resolution of spectral features due to greater 1s core–hole lifetime broadenings (4.14 and 4.52 eV natural broadenings, respectively)^44^ and the appearance of VtC features on the high-energy tail of the Kβ_2_ emission line (Figure 1).^12,45–47^ For example, while the VtC Kβ″ line for 3d transition metal complexes has allowed for ligand identification in homogeneous and heterogeneous systems,^24,37,39^ bond activation quantification,^27^ and differentiation between CO and hydrocarbon ligand-based transitions,^48^ the broadening of the corresponding line in Mo carbonyl, chloride, silicide, and sulfide compounds has prevented resolution of transitions from ns ligand orbitals to metal 1s orbitals.^12,46^ For later 4d transition metals and 5d transition metals, spectral broadening is expected to become even larger (6.24 eV natural broadening for Pd)^44^ and further limit detection of informative spectral features. Another technique for directly interrogating valence orbitals of 4d and 5d transition metal compounds is ultraviolet photoelectron spectroscopy (UPS), which measures the kinetic energies of electrons ejected from valence orbitals using ultraviolet light.^49–51^ However, UPS generally requires ultrahigh vacuum and thin layers of conducting materials for measurements and thus has more limited applications for in situ spectroscopy.^50^ As such, the development of alternative VtC XES methods, such as L-shell 4d-to-2p XES, is of interest.

**Figure 1:**
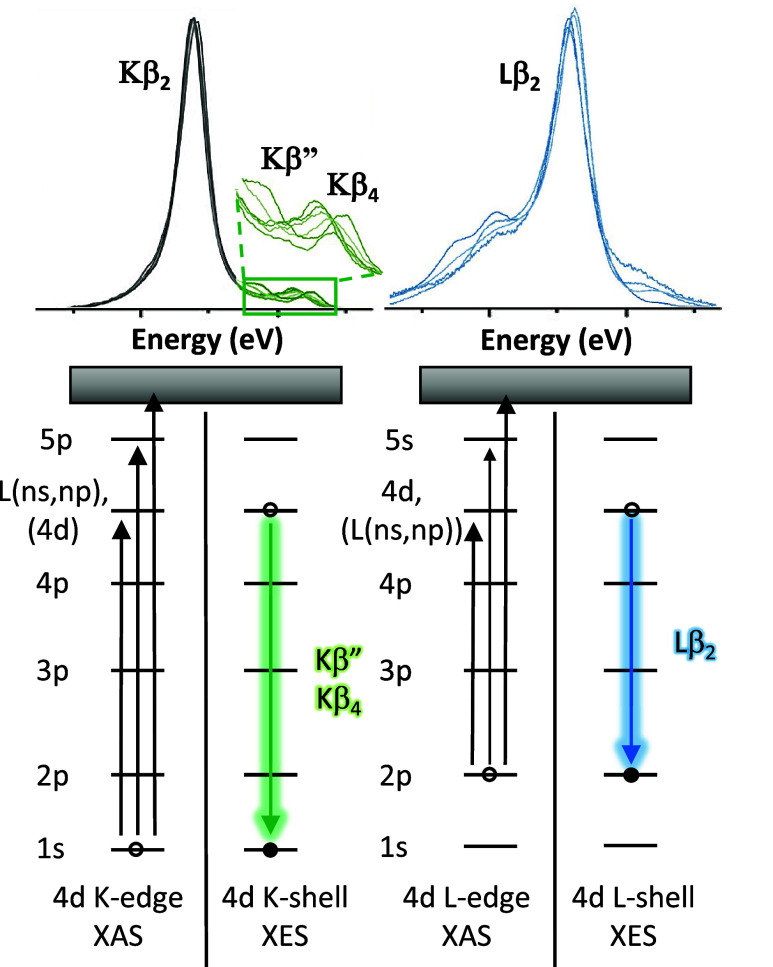
Example XES spectra (top)^12,13^ and energy level diagrams (bottom) for the K- and L-edge absorption and emission processes for the 4d transition metals Mo (left) and Ru (right). The top left part of the figure was partially reproduced with permission from the Creative Commons Attribution License from ref 12. Copyright 2020 The Authors. Published by Wiley-VCH Verlag GmbH & Co. KGaA. The top right part of the figure was partially reproduced from ref 13. Copyright 2020 American Chemical Society.

4d-to-2p XES takes advantage of dipole-allowed transitions from metal-based d-orbitals to metal-based p-orbitals, larger orbital overlap integrals, and decreased core–hole lifetime broadening from p-orbitals resulting in increased transition intensities compared to K-shell XES.^2–4,10,11,23^ In the case of Ru 4d-to-2p XES, for example, a combined experimental and DFT approach demonstrated an increase in the intensity of transitions involving π-bonding orbitals in a [Ru­(CN)_6_]^4–^ complex,^13^ whereas similar π-interactions were not detected in K-shell XES spectra of Mo carbonyl complexes.^12^ This study and additional studies of Ru compounds highlight the promise of L-shell XES to increase the resolution for 4d transition metal XES spectroscopy. L-shell 5d-to-2p XES of Pt complexes has also been reported, but clear differences in the spectral features due to changes in metal oxidation state and ligand covalency and identity were not observed unless resonant excitation was employed.^52^ Aside from a few more nonresonant nd-to-2p XES studies of Ru complexes,^53,54^ most studies focus on 2p-to-nd resonant XES (RXES), but for only Ru and Pt.^52,53,55,56^ While RXES experiments can provide a wealth of information about metal species, the complexity of the experimental requirements and the interpretation of the data make nonresonant XES experiments more appealing and readily accessible. The limited number of L-shell VtC XES experiments and the number of metals investigated make further studies necessary to fully understand the utility of this technique for the analysis of 4d and 5d transition metals in both materials characterization and in situ catalysis studies.

One ubiquitous 4d transition metal catalyst is Pd, which has applications in industrial and laboratory-based hydrogenation,^57,58^ cross-coupling, and oxidation reactions^59–67^ and is of interest for developing fuel cell technologies,^68,69^ electrochemical synthesis,^70–72^ and the direct synthesis of H_2_O_2_.^73–75^ Despite the long history and numerous studies of Pd catalysts, including many involving K-edge XAS, a detailed mechanistic understanding of many Pd catalytic reactions and their structure–function relationships are still lacking, though this information is essential to guide rational catalytic design.^59,60,62,64^ Among the various uses of Pd in homogeneous and heterogeneous catalysis, its use in oxidation reactions is a major application.^59–64^ The use of O_2_ as the oxidant in these reactions is desirable due to its abundance, low cost, and reduced toxicity.^61,62,69,70,76^ Also of interest is the development of catalysts that can use O_2_ as a reactant, being activated on Pd catalytic centers and then reacting further with hydrocarbons, protons, and other reactants.^62,73,74,77^ As such, an L-shell 4d-to-2p XES study of a series of well-defined Pd complexes with various O-containing ligands is of interest to evaluate how different Pd oxidation states, coordination environments, and ligands affect spectral features, whether these features can be predicted with density functional theory (DFT) calculations, and whether 4d-to-2p XES can differentiate among intermediates formed during catalytic reactions in homogeneous, nanoparticle, and electrode systems, among others.

The Pd complexes investigated by 4d-to-2p XES are shown in Figure 2.^78–81^ The series includes Pd bis-NHC (NHC = N-heterocyclic carbene) complexes that vary in oxidation state and axial ligand identity (**1**–**4**), as well as coordination geometry (**5**). Monometallic and bimetallic complexes with pyrazole or chloride ligands (**6**–**8**) were also studied to further understand how the ligand identity and the coordination sphere modulate spectral features. DFT calculations were performed on this series of complexes to determine the origin of the spectral features and to evaluate the ability of DFT calculations to predict Pd 4d-to-2p XES spectral features. Also presented are 4d-to-2p XES measurements of Pd nanoparticles (NPs) to understand the feasibility of using 4d-to-2p XES to investigate Pd systems in heterogeneous catalysis.

**Figure 2:**
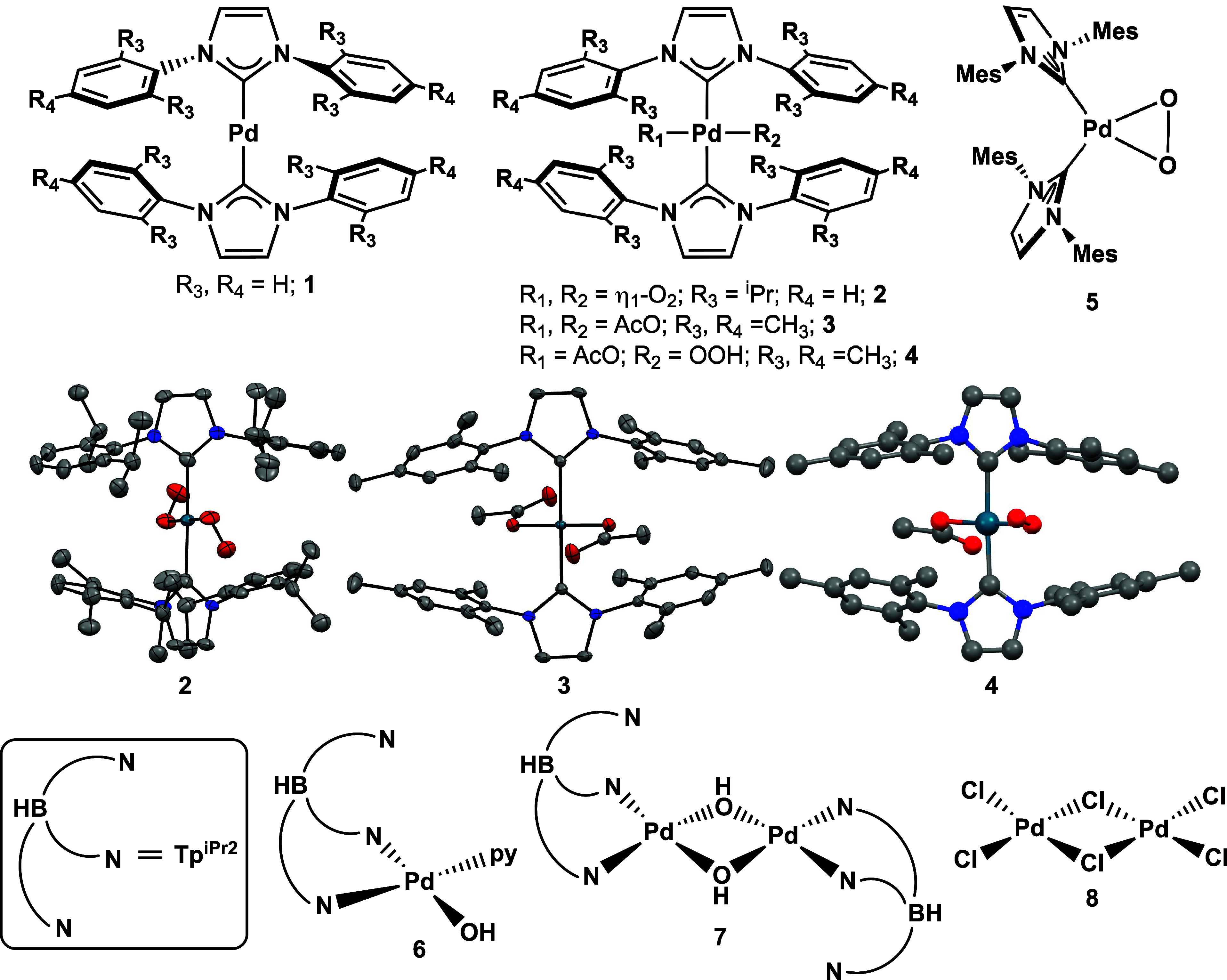
Pd complexes (**1**–**8**) investigated by 4d-to-2p XES and DFT (^i^Pr = isopropyl; AcO = acetate; Mes = mesityl; Tp^iPr2^ = hydrotris­(3,5-diisopropylpyrazolyl)­borate; py = pyridine). Crystal structures are shown for **2** and **3** (middle).^79,80^ Thermal ellipsoids are plotted at the 50% probability level, and hydrogens and solvent atoms have been omitted for the sake of clarity. The DFT-optimized structure for **4** is also shown (middle), but no crystal structure has been published. The CCDC deposition numbers for molecular compounds **1**–**3** and **5**–**7** are 836258, 800883, 249608, 249607, 1243235, and 1243236, respectively.

## Results and Discussion

The first set of compounds selected for this study consisted of PdNHC_2_ complexes with different Pd oxidation states, oxygen-based ligands, and coordination geometries (**1**–**5** (Figure 2)). Compound **1** is a Pd(0) complex, while **2**–**5** are Pd­(II) species with anionic superoxo, hydroperoxo, and/or acetate ligands, or a peroxo ligand. Based on the single-crystal X-ray diffraction structures, these complexes show similar Pd–NHC and Pd–O bond distances (Table 1), with **1** having the shortest Pd–NHC bond distances and compound **5** displaying the greatest ligand bond activation and the second shortest Pd–NHC bond lengths.^79–81^

**Table 1:** Summary of Bond Lengths (angstroms) for **1–5**

	**1**	**2**	**3**	**4** ^a^	**5**
Pd–O	N/A	2.011	2.012	2.046	2.010
		2.013	2.012	1.998	2.010
Pd–NHC	2.022	2.059	2.043	2.053	2.041
	2.025	2.065	2.043	2.055	2.027
O–O or (O–C)	N/A	1.314	(1.280)	(1.282)	1.443
		1.340	(1.280)	1.437	

a The bond lengths from the DFT-optimized structure of **4** are listed as there is no published crystal structure available.

The 4d-to-2p XES spectra of compounds **1**–**5** are shown in Figure 3. The spectra show clear differences in the energy of the maximum of the Lβ_2_ line and the energy and intensity of the shoulder feature at ∼3169 eV. The complex with the lowest energy maximum is **1**, consistent with a Pd(0) oxidation state, followed by **2**, suggesting a more reduced Pd center compared to those of **3**–**5**. The intensity of the shoulder feature at ∼3169 eV increases upon going from **1** to **5**. Thinking in a simplistic molecular orbital diagram picture, the lower-energy transitions should originate from ligand-based valence orbitals, while higher-energy transitions should involve the filled Pd-based d-orbitals and any higher-lying filled antibonding ligand orbitals. Regardless of the local symmetry of **1**–**5**, all transitions from Pd s- or d-orbitals to the Pd 2p core–hole will be dipole allowed. In the *D*_2*h*_ limit, transitions from p-orbitals are dipole forbidden, but a decrease in symmetry to *D*_2_, which eliminates inversion symmetry, will create dipole-allowed transitions from p-orbitals. Considering the binding modes of the ligands in **1**–**5** (Figure 2), the transition intensities will therefore most likely be governed by the amount of Pd orbital character in the relevant orbitals and thus, for the ligand-to-metal charge-transfer (LMCT) transitions, the strength of the metal–ligand interactions. This is consistent with the spectra as the complexes with the lowest oxidation states based on the energy of the maximum of the Lβ_2_ line (**1** and **2**) and the longest Pd–NHC bonds (**2**) result in the least intense transitions for the shoulder feature at ∼3169 eV. The complex with the highest-intensity shoulder feature at ∼3169 eV (**5**) displays a Pd­(II) oxidation state, the strongest ligand activation, and the second-shortest Pd–NHC bond lengths, consistent with increased ligand orbital mixing.

**Figure 3:**
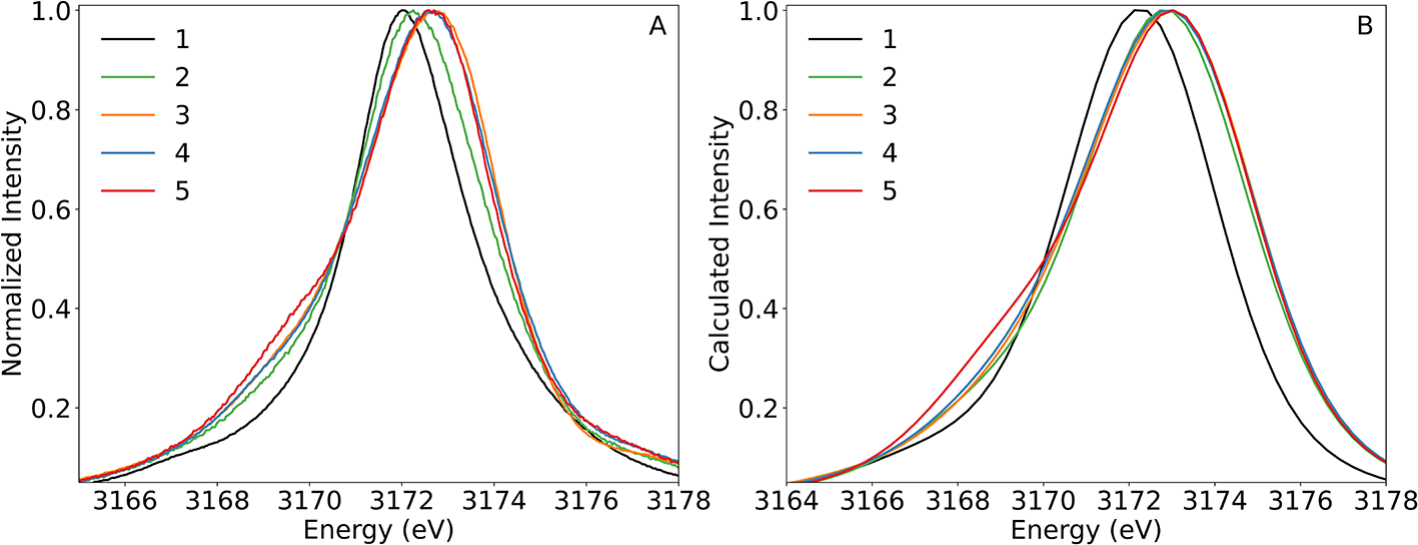
(A) 4d-to-2p XES spectra of compounds **1**–**5** normalized to the maximum intensity of each spectrum and (B) DFT-calculated 4d-to-2p XES spectra of compounds **1**–**5** normalized to the maximum intensity of each curve. An energy shift of 22 eV and a Voigt broadening of 2.46 eV (fwhm) were applied to the DFT-calculated spectra, consistent with the convolution of the spectrometer resolution (1 eV) and the natural broadening of the tabulated L_3_ line for Pd (2.25 eV), which is based on the value from ref 44.

To better understand the effects of Pd ligand interactions and orbital mixing on the transitions arising from the two distinct spectral regions (3166–3171 and 3171–3174 eV), DFT calculations were performed. The optimized structures of **1**–**3** and **5** agree well with the reported crystal structures (Tables S1 and S2), and the calculated XES spectra nicely reproduce the trends in the intensity and energy of the major spectral features (Figure 3). The broadening applied to the calculated XES spectra, determined from the convolution of the spectrometer resolution and the natural broadening of the tabulated L_3_ line for Pd, is greater than that apparent in the experimental spectra, indicating an overestimation of spectral broadening from this method. The red-shift in the energy of the most intense spectral feature of **2** from those of **3**–**5** is, however, underestimated by the calculations. This is likely due to the more complex open-shell nature of **2**, which is more difficult to treat with DFT. Analyzing the simplest molecule (**1**), the transition that contributes most to the intensity of the feature at ∼3167 eV involves two ligand-based orbitals resulting from σ interactions between the p_*z*_-orbitals of the NHC ligands and the d_*z* ^2^_ Pd orbital (Figure 4). The most intense feature at ∼3172 eV results from transitions from the Pd d-based orbitals, where the Pd d_*xy*_ and d_*x* ^2^–*y* ^2^_ show no significant mixing with the NHC ligand p-orbitals (Figure 4). A more detailed analysis of the factors that affect the intensity of the transitions reveals that the percent Pd d-orbital contribution to the corresponding orbitals scales linearly with the intensity of the transition (Figure S1). No other orbital contributions from Pd or the NHC ligands or a combination of orbital contributions were found to correlate systematically with the intensities of the transitions. This underscores the importance of considering Pd d-orbital mixing when predicting transition intensities. This is expected, as in the case of 4d-to-2p XES of complexes with symmetry lower than *D*_2*h*_, all transitions are dipole allowed and the intensities primarily depend on the overlap integral between the donor orbital and the Pd 2p core orbitals.

**Figure 4:**
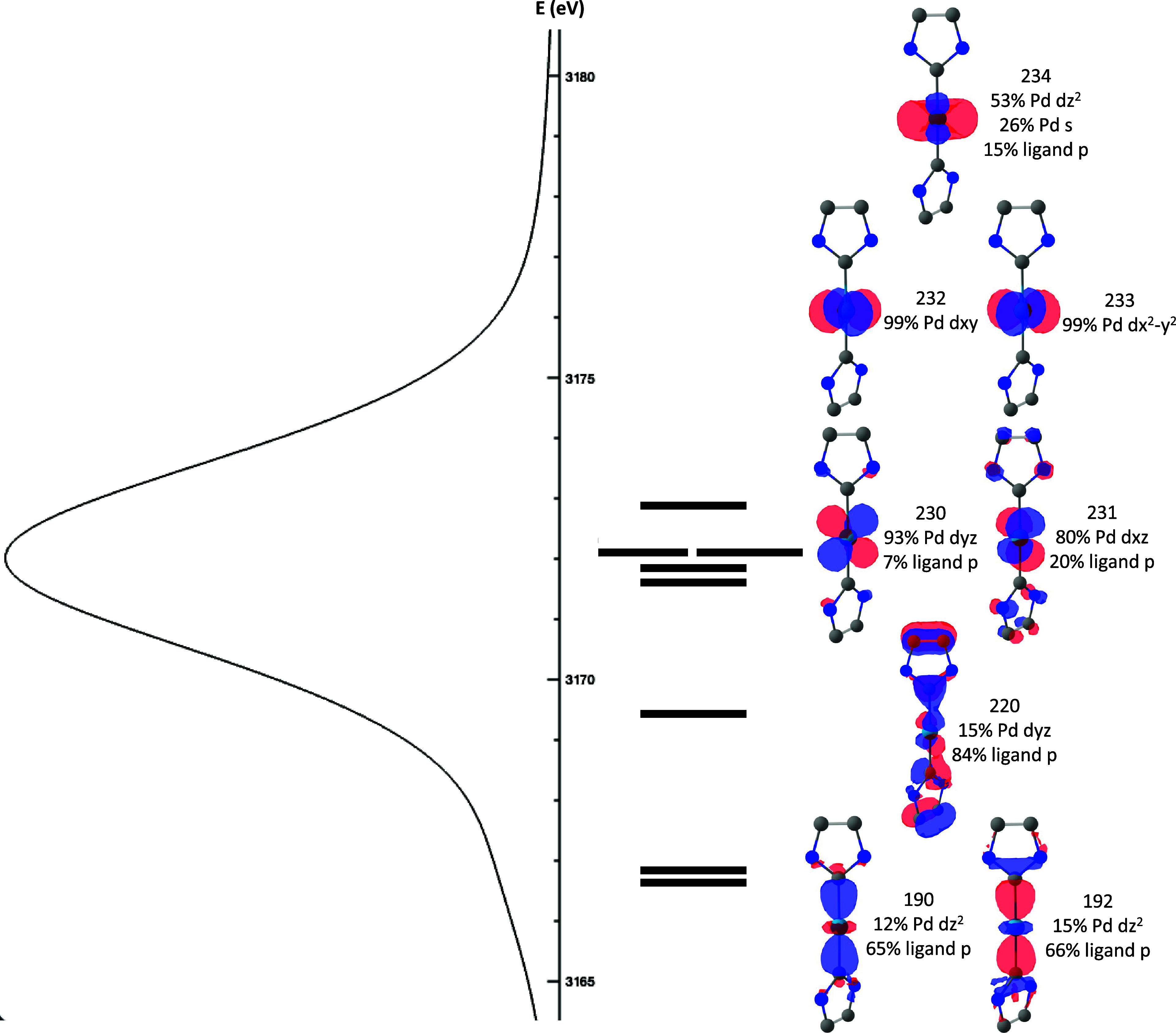
Molecular orbitals corresponding to the most intense transitions for the calculated 4d-to-2p XES spectra of **1**. A truncated structure of **1** is shown for the sake of clarity.

Continuing the DFT analysis for compounds **2**–**5** reveals findings similar to those of **1**. The lower-energy and lower-intensity transitions between 3166 and 3171 eV primarily involve LMCT from orbitals resulting from the σ interactions between the Pd and ligands, while the higher-energy, higher-intensity transitions involve primarily Pd d-based orbitals (Figures S2–S5). Unlike in **1**, these higher-energy transitions now include significant contributions from σ and π interactions of Pd with bonding and antibonding ligand orbitals (Figures S2–S5). The dependence of the transition intensities on the percent Pd d-character in the corresponding orbitals follows the same linear dependence as in **1** (Figure S1). Upon comparison of the compositions of the ligand-based orbitals that contribute the most to the shoulder feature at ∼3169 eV, the percent Pd d-orbital character increases from **1** to **5**, tracking with the intensity of this feature (Figure 4 and Figures S2–S5). In **5**, there are additional relatively intense transitions at lower energies originating from both σ bonding interactions with the NHC and the O_2_ ligands as well as π-bonding interactions with the O_2_ ligand that contribute to the more prominent shoulder feature at around 3169 eV (Figure S5 and Figure 5). In this case, **5** is able to utilize d_*x* ^2^–*y* ^2^_, d_*xy*_, d_*xz*_, and d_*z* ^2^_ orbitals to create multiple interactions with the O_2_ and the NHC ligands, resulting in four orbitals with relatively large amounts of Pd d-orbital mixing. Complexes **2**–**4**, on the other hand, do not have orbitals with optimal symmetry or energy to significantly overlap with other ligand orbitals. The combined data for complexes **1**–**5** indicate that increased mixing of Pd d-orbitals with ligand orbitals and greater ligand activation results in more intense transitions between 3166 and 3171 eV, resulting in more intense shoulder features (Figure 5).

**Figure 5:**
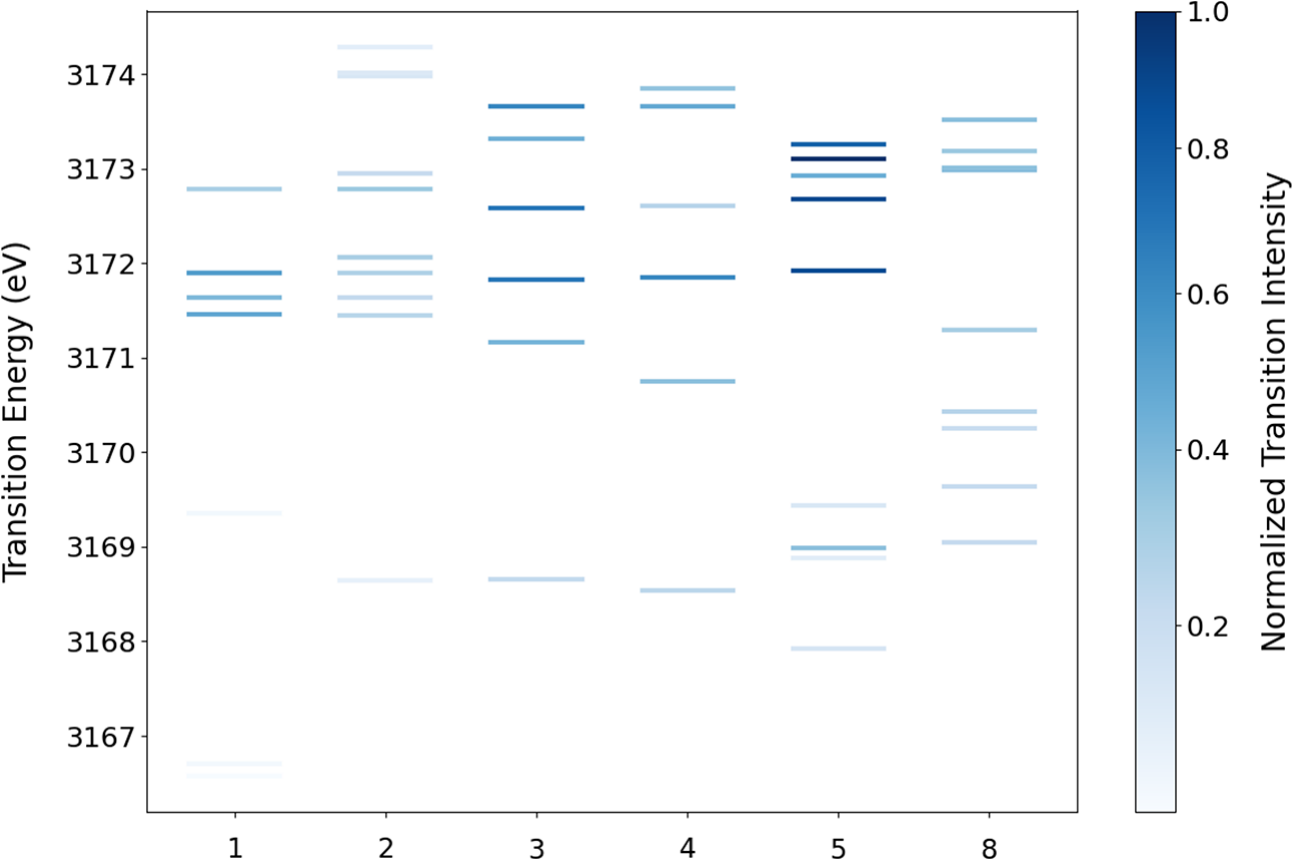
Plot of the transition energies for major transitions for **1**–**5** and **8** with the transition intensity indicated by different shadings of the lines for each transition.

In contrast to the more thoroughly studied K-shell VtC XES for 3d transition metals, 4d-to-2p XES should have increased transition intensities due to the dipole-allowed nature of the transitions and increased metal orbital character. Calculations of Pd 4d-to-1s XES spectra were therefore carried out and indicate a more than 300-fold decrease in transition intensities compared to those for 4d-to-2p XES for **1** and **5** (Figures S6 and S7). In addition, a broadening of the calculated Pd 4d-to-1s XES spectra based on the natural broadening for Pd (6.25 eV)^44^ results in a curve with a single peak (Figures S6 and S7), indicating that K-shell spectroscopy for Pd would provide limited information about ligand identity and transformation.

To investigate how the spectral features change with ligands similar to but distinct from the NHC ligands of **1**–**5**, as well as to compare bimetallic and monometallic complexes, **6** and **7** (Figure 2) were also synthesized, and XES measurements together with DFT computations were performed. The XES spectra show differences in the feature at ∼3169 eV as in complexes **1**–**5**, and the DFT-calculated spectra again show agreement with the experimental spectra, highlighting the ability of DFT to correctly predict trends in the intensity of features in PdO_*x*_H_*y*_ complexes (Figure 6). Additionally, the dependence of the transition intensities on the % Pd d-character in the corresponding orbitals is the same as for **1**–**5**, suggesting that for similar ligands and similar or lower complex symmetries the dependence of the transition intensities on the percent Pd d-character is the same (Figure S1).

**Figure 6:**
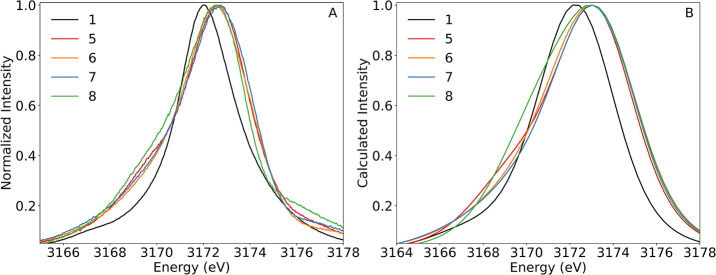
(A) 4d-to-2p XES spectra of compounds **1** and **5**–**8** normalized to the maximum intensity of each spectrum and (B) DFT-calculated 4d-to-2p XES spectra of compounds **1** and **5**–**8** normalized to the maximum intensity of each spectrum. An energy shift of 22 eV and a Voigt broadening of 2.46 eV (fwhm) were applied to the DFT-calculated spectra, consistent with the convolution of the spectrometer resolution (1 eV) and the natural broadening of the tabulated L_3_ line for Pd (2.25 eV), which is based on the value from ref 44.

Considering a Pd complex with σ- and π-donating chloride ligands and a higher symmetry (*D*_2*h*_) imposed by an extended solid structure, XES measurements of Pd_2_Cl_6_ (**8**) were also performed and DFT computations were again able to predict spectral features (Figure 6). **8** displays a broader and more intense shoulder feature centered at ∼3170 eV. DFT analysis of the highest-intensity transitions for **8** reveals transitions from a number of orbitals with large Pd d-orbital contributions (Figure S8). The symmetry of the complex, the number of filled chloride valence orbitals, and the energy of the chloride orbitals allow for multiple Pd–Cl interactions that create a number of intense transitions spread out in energy between ∼3167 and ∼3171 eV and, thus, an intense and broad shoulder feature (Figure 5). For this complex, the linear relationship of the transition intensity and the percent Pd d-character is modulated as compared to those of **1**–**7** (Figure S1). This difference can, in part, be explained by the fact that the oscillator strength of a transition is a product of both the overlap integral of the donor and acceptor orbitals and the intrinsic dipole character of the transition.^22,82^ The dependence of the transition intensities on the intrinsic dipole integrals of **8** also differs from that of **1**–**7** (Figure S9) and is likely due to a combination of factors such as the elimination of contributions from p-to-p transitions due to the higher symmetry of **8** (*D*_2*h*_) compared to **1**–**7** (*C*_2*v*_ or lower), as well as the decreased covalency of the chloride ligands compared to the ligands of **1**–**7**.

The results from the 4d-to-2p XES measurements of **1**–**8** indicate that the increased strength and number of metal ligand interactions in molecular complexes result in resolvable intensity increases of shoulder features between ∼3166 and ∼3171 eV in XES spectra. To understand if such an increase in intensity due to ligand binding and activation on Pd catalysts could be resolved in Pd NP samples, XES analysis of 1% Pd NP loaded on a carbon support was performed. The NP samples were measured in as-prepared (**9**) and H_2_-reduced (**10**) states. The XES data of these two samples show minor differences in the line shapes but a 0.2 eV difference in the intensity-weighted average energy (IWAE), consistent with the NP samples in different oxidation states (Figure 7). The X-ray photoelectron spectra (XPS) (Figures S10 and S11) of **9** and **10** indicate the presence of mostly Pd­(II) at the surface of the 1% Pd NPs, with an only 35% reduction in surface Pd­(II) to Pd(0) after treatment with H_2_, consistent with the relatively small shift in IWAE observed in the XES spectra, a bulk spectroscopy technique. Compared to **5**, the NP samples show a general broadening, but the absence or reduced intensity of the shoulder feature at ∼3169 eV. These results are promising in that O_2_ binding and activation on Pd NP surfaces could cause an increase in intensity between ∼3167 and ∼3171 eV that could be detected by 4d-to-2p XES, allowing for a probe of the activity of different Pd catalysts in situ.

**Figure 7:**
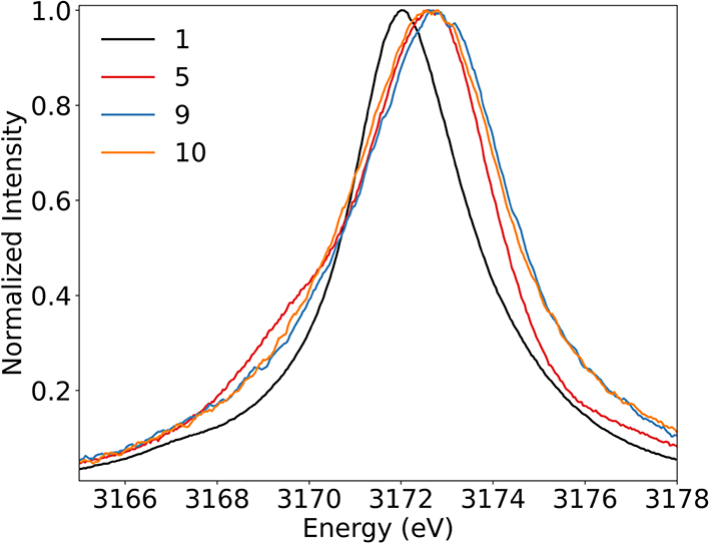
4d-to-2p XES spectra of **1**, **5**, **9**, and **10** normalized to the maximum intensity of each spectrum.

## Conclusions

This study highlights the ability of Pd 4d-to-2p XES to resolve differences in spectral features in a series of PdO_*x*_H_*y*_ complexes due to differing Pd oxidation states, ligand environments, and coordination geometries. A lower-energy feature between 3166 and 3172 eV has been identified that is attributed dominantly to transitions from valence ligand p-orbitals. In particular, this feature is sensitive to the strength of Pd–ligand interactions and the extent of bond activation in ligands. DFT calculations are reliable in predicting the spectral features of these complexes and can aid in the identification of unknown Pd species formed in chemical reactions and during catalysis. The 4d-to-2p XES spectra of Pd NP samples exhibit absent or low-intensity shoulder features, indicating the possibility of resolving the appearance or increase in the intensity of this feature upon reactant binding and activation. These results highlight the promise of 4d-to-2p XES to provide new insight into Pd systems for which characterization is lacking, including ex situ and operando applications in both homogeneous and heterogeneous Pd catalysts.

## Methods

### Complex Synthesis

PdCl_2_ was purchased from Sigma-Aldrich and used without further purification. (κ^2^-Tp^iPr_2_^)­(OH)­(py)Pd (Tp^iPr_2_^ = hydrotris­(3,5-diisopropylpyrazolyl)­borate; py = pyridine), (κ^2^-Tp^iPr_2_^Pd)_2_(μ-OH)_2_, PdIPr_2_ (IPr = 1,3-bis­(2,6-diisopropylphenyl)-1,3-dihydro-2*H*-imidazol-2-ylidene), PdIPr_2_(η_1_-O_2_)_2_, PdIMes_2_(AcO)_2_ (IMes = 1,3-bis­(2,4,6-trimethylphenyl)-1,3-dihydro-2*H*-imidazol-2-ylidene; AcO = acetate), PdIMes_2_(AcO)­(OOH), and PdIMes_2_(η_2_-O_2_) were synthesized according to established literature procedures.^78–81^

### XES Measurements

All samples were prepared in a N_2_-filled glovebox and stored under liquid nitrogen until measurement. All samples were measured in the solid state at 30 K. The pure solids were ground to a fine powder and packed into 1 mm thick aluminum sample holders. The back and irradiated side of the cell were covered with 13 μm Kapton tape.

Pd Lβ_2_ XES data were collected at the PINK tender X-ray beamline^83^ at BESSY II. The spectra were collected using an in-house-designed energy dispersive vacuum von Hamos spectrometer. A Ge(220) 1 mm striped crystal with a bending radius (*R*) of 247 mm dispersed incoming fluorescence radiation onto a 1 in. GreatEyes CCD detector with a 26 μm × 26 μm pixel size (256 × 1024 pixels). The CCD detector accepted fluorescent radiation in a 3160–3195 eV energy window that corresponds to Bragg angles of θ = 78.8–75.9°. The spectrometer resolution was about 1 eV.

The samples were cooled to 30 K using helium as the exchange gas. The excitation energy was set to 4000 eV using a multilayer monochromator (Δ*E* of 80 eV). The beam size at the sample position was 30 μm × 500 μm fwhm (*V* × *H*) with a photon flux of ∼4 × 10^13^ photons/s. In order to reduce the amount of radiation damage, the data were collected with continuous sample motion at a rate of 150 μm/s, resulting in an effective sample exposure of 0.2 s per spot. Each pass took approximately 5 min. Then, the scanning procedure was repeated. The typical measurement time per sample was 10 min or less.

To properly calibrate the spectrometer, at least two emission lines are needed. For the spectrometer geometry used, the Pd Lβ_2_ line (at the 220 reflection) and the Fe Kα_2_ line (at the 440 reflection) were within the relatively small field of view of the spectrometer and thus chosen for the calibration. For the energy calibration procedure, Pd and Fe foils were measured in the same configuration and calibrated to the Pd Lβ_2_ line at 3171.79 eV and the Fe Kα_2_ line at 6390.84 eV.^84^ While the Pd Lβ_2_ spectra were collected using the Ge(220) reflection, the Fe Kα_2_ lines were collected with the Ge(440) reflection of the same crystal without any rearrangements of the beamline optics or the spectrometer. Obtaining the Fe Kα_2_ XES spectra required a higher excitation energy that was achieved with second-order radiation at *E* = 8000 eV. Positions of the Pd Lβ_2_ and Fe Kα_2_ lines on the detector were defined by the center of mass of the line. The energies were translated into Bragg angles and a fit with a tangential function. After performing the energy calibration procedure, the XES data were normalized to the maximum intensity of each spectrum.

### Theoretical Calculations

All calculations were performed with the ORCA version 5.03 quantum chemistry software package^85^ using the PBE0 functional^86^ and the ZORA-def2-TZVP basis set^87,88^ for all atoms except Pd, for which the SARC-ZORA-TZVP basis set^89^ was used. Scalar relativistic effects were included using ZORA. The auxiliary basis set was automatically generated using the keyword AutoAux.^90^ X-ray crystal structures were used as starting points for geometry optimizations for **1**–**3** and **5**–**8**.^78–81,91^ For **4**, the crystal structure of **3** was used as a starting point for which one of the acetate ligands was substituted with a hydroperoxo ligand. The conductor-like polarizable continuum model (CPCM) was used for charge compensation in all calculations.^92^ XES calculations were performed using previously established procedures^32^ and the RIJCOSX exchange algorithm for increased computational efficiency.^93^ Example input files with additional information are provided in the Supporting Information. Orbitals were generated using the orca_plot utility tool and visualized with ChemCraft. Spectra and MO orbitals diagrams were generated with the orca_mapspc utility tool and MOAnalyzer.^94^ An energy shift of 22 eV and a Voigt broadening of 2.46 eV (fwhm) were applied to the DFT-calculated spectra, consistent with the convolution of the spectrometer resolution (1 eV) and the natural broadening of the tabulated L_3_ line for Pd (2.25 eV).^44^ Example input files and optimized *xyz* coordinates for compounds **1**–**8** are provided in the Supporting Information.

### Nanoparticle Synthesis

Monometallic 1% Pd/C was prepared on a commercially available activated carbon support (Norit ROX 0.8) on a weight basis by a sol-immobilization procedure based on a methodology previously reported in the literature,^95^ which has been shown to result in enhanced precious metal dispersion by limiting particle growth.^96^ The procedure to produce the 1% Pd/C catalyst (2 g) is outlined below.

An aqueous solution of PdCl_2_ (3.333 mL, [Pd] = 6.0 mg mL^–1^, Merck) was added to deionized water (800 mL) under vigorous stirring at room temperature. The resulting solution was allowed to stir for 2 min prior to the addition of poly­(vinyl alcohol) (PVA) (2.40 mL, 1 wt %, MW = 9000–10 000 g mol^–1^, 80% hydrolyzed, Merck) such that the metal:PVA weight ratio was 1:1.2. The resulting solution was stirred for 2 min prior to the addition of a freshly prepared solution of NaBH_4_ (9.397 mL, 0.1 M, Merck) such that the NaBH_4_:Pd molar ratio was 5:1. Upon the addition of NaBH_4_, the mixture turned dark brown and was stirred vigorously for an additional 30 min followed by the addition of Norit ROX 0.8 (1.98 g, ground to obtain a 100–140 mesh). The solution was acidified to pH 1 via the addition of H_2_SO_4_ (>95%, Fischer Scientific) and allowed to stir for a further 1 h. The need for acidification of the catalyst synthesis solution can be related to the kinetics of nanoalloy immobilization where, through acidification, it is possible to promote deposition of the PVA-encapsulated metal species and therefore achieve good control over nanoparticle size. Following this, the suspension was filtered under vacuum, washed thoroughly with distilled water until the pH of the washings was neutral, and then dried (110 °C, 16 h, static air). The resulting material was subsequently ground and calcined (400 °C, 3 h, 10 °C min^–1^, and static air).

The H_2_-reduced 1% Pd/C material was prepared in a N_2_-filled glovebox by flowing 100% H_2_ over the as-prepared 1% Pd/C material for 30 min in a vial, sealing the vial, and letting further reduction occur over 24 h under 1 bar of H_2_. The XES sample was then prepared in a N_2_-filled glovebox as described above.

The as-prepared 1% Pd/C and the H_2_-reduced 1% Pd/C materials were characterized with transmission electron microscopy (TEM) and X-ray photoelectron spectroscopy (XPS), the details of which are provided in the Supporting Information.

## Supplementary Material



## Data Availability

All other relevant data generated and analyzed during this study, which include experimental, spectroscopic, and computational data, are available in the Edmond Open Research Data Repository at 10.17617/3.G8CBHT and in the Supporting Information.

## Notes

Additional figures, tables with calculated bond lengths, XPS and TEM measurement details, XPS and TEM data, example input files for DFT calculations, and optimized *xyz* coordinates for all complexes (PDF)
