# Supplementary material for: Evaluating Palladium 4d-to-2p X‑ray Emission Spectroscopy for Characterizing Catalytically Relevant Species
Source: Inorg Chem. 2026 Jan 9;65(3):1801–11. doi: 10.1021/acs.inorgchem.5c04266 (PMC12848978; doi:10.1021/acs.inorgchem.5c04266)
Supplement: Supplementary file 1 [file ic5c04266_si_001.pdf]

Supporting Information for

Evaluating Palladium 4d-to-2p X-ray Emission Spectroscopy for Characterizing Catalytically Relevant Species

Anna G. Scott[a], Sergey Peredkov[a], Angeles Lopez-Martin[b], Richard J. Lewis[b], Graham J. Hutchings[b], Serena DeBeer[a,*]

*serena.debeer@cec.mpg.de

a Max Planck Institute for Chemical Energy Conversion, D-45470 Mülheim an der Ruhr, Germany
b Max Planck–Cardiff Centre on the Fundamentals of Heterogeneous Catalysis FUNCAT, Cardiff Catalysis Institute, School of Chemistry, Cardiff University, Cardiff, CF24 4HQ, United Kingdom

Table of Contents

**Table S1**	S2
**Table S2**	S2
**Figure S1**	S3
**Figure S2**	S3
**Figure S3**	S4
**Figure S4**	S5
**Figure S5**	S6
**Figure S6**	S7
**Figure S7**	S8
**Figure S8**	S9
**Figure S9**	S10
**XPS Measurement Details**	S10
**Figure S10**	S11
**Figure S11**	S11
**TEM Measurement Details**	S11
**Figure S12**	S12
**Figure S13**	S12
**Input file examples for geometry optimizations and the calculation of XES spectra**	S13
**Optimized xyz coordinates for all complexes**	S14
**References**	S29

Table S1: Summary of Bond Lengths for 1-3 and **5 (SC-XRD)**

Bond Lengths (Å)	**1**	**2**	**3**	**5**
Pd-O	N/A	2.011<br>2.013	2.012<br>2.012	2.010<br>2.010
Pd-NHC	2.022<br>2.025	2.059<br>2.065	2.043<br>2.043	2.041<br>2.027
O-O or (O-C)	N/A	1.314<br>1.340	(1.280)<br>(1.280)	1.443
Bond lengths[a]		O_2: 1.21	(OAc^-: 1.26)	O_2: 1.21

[a]Bond lengths from Ref. 1.[1]

Table S2: Summary of Bond Lengths for 1-5 (DFT)

Bond Lengths (Å)	**1**	**2**	**3**	**4**	**5**
Pd-O	N/A	2.011<br>2.012	2.022<br>2.022	2.046<br>1.998	1.991<br>1.992
Pd-NHC	2.039<br>2.045	2.072<br>2.072	2.060<br>2.060	2.053<br>2.055	2.047<br>2.049
O-O or (O-C)	N/A	1.292<br>1.292	(1.284)<br>(1.284)	(1.282)<br>1.437	1.377
Bond lengths[a]		O_2: 1.21	(OAc^-: 1.26)	(OAc^-: 1.26)<br>HOOH: 1.48	O_2: 1.21

[a]Bond lengths from Ref. 1.[1]

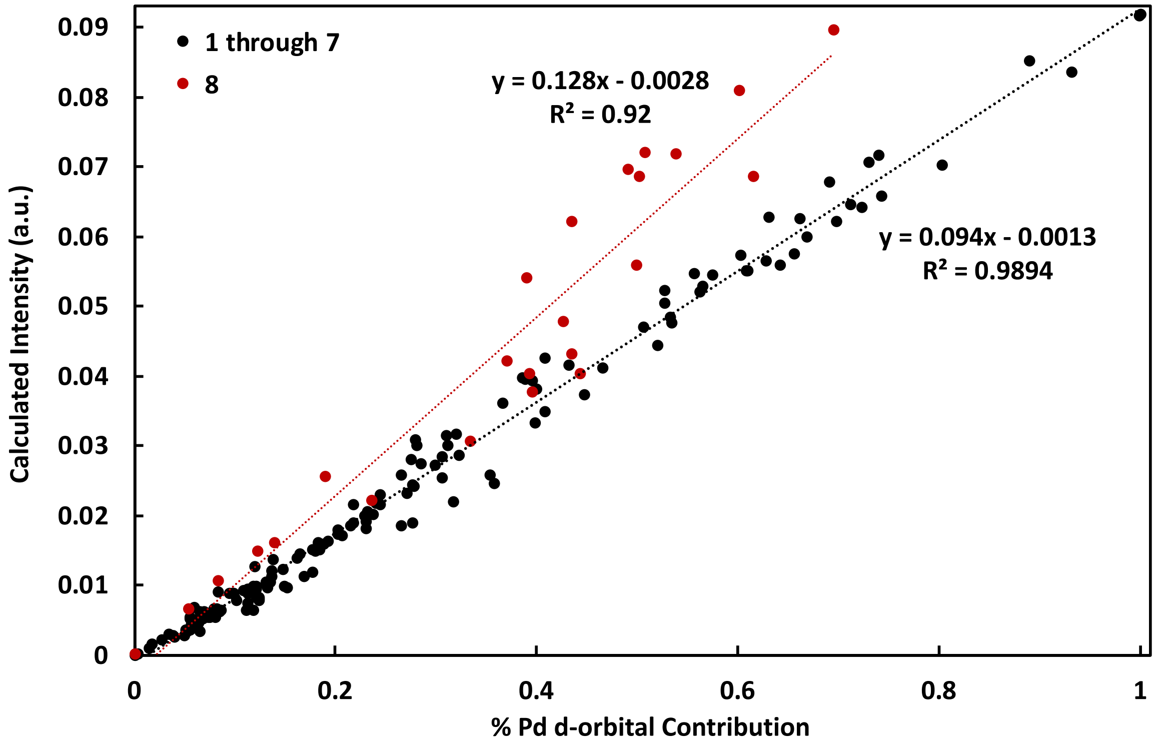

Figure S1: Trend of % Pd d-orbital contribution to selected orbitals and intensity of corresponding transition for **1**-**8**.

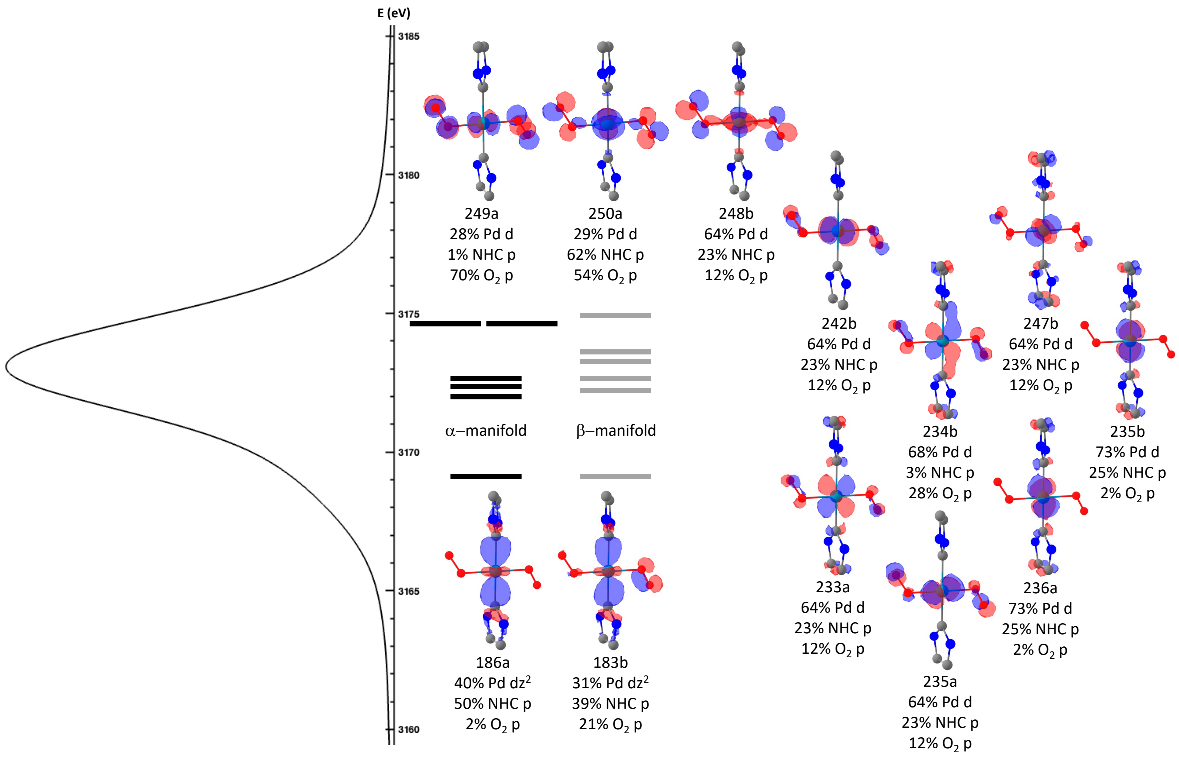

Figure S2: Molecular orbitals corresponding to most intense transitions calculated for 4d-to-2p XES spectra of **2**. A truncated structure of **2** is shown for clarity.

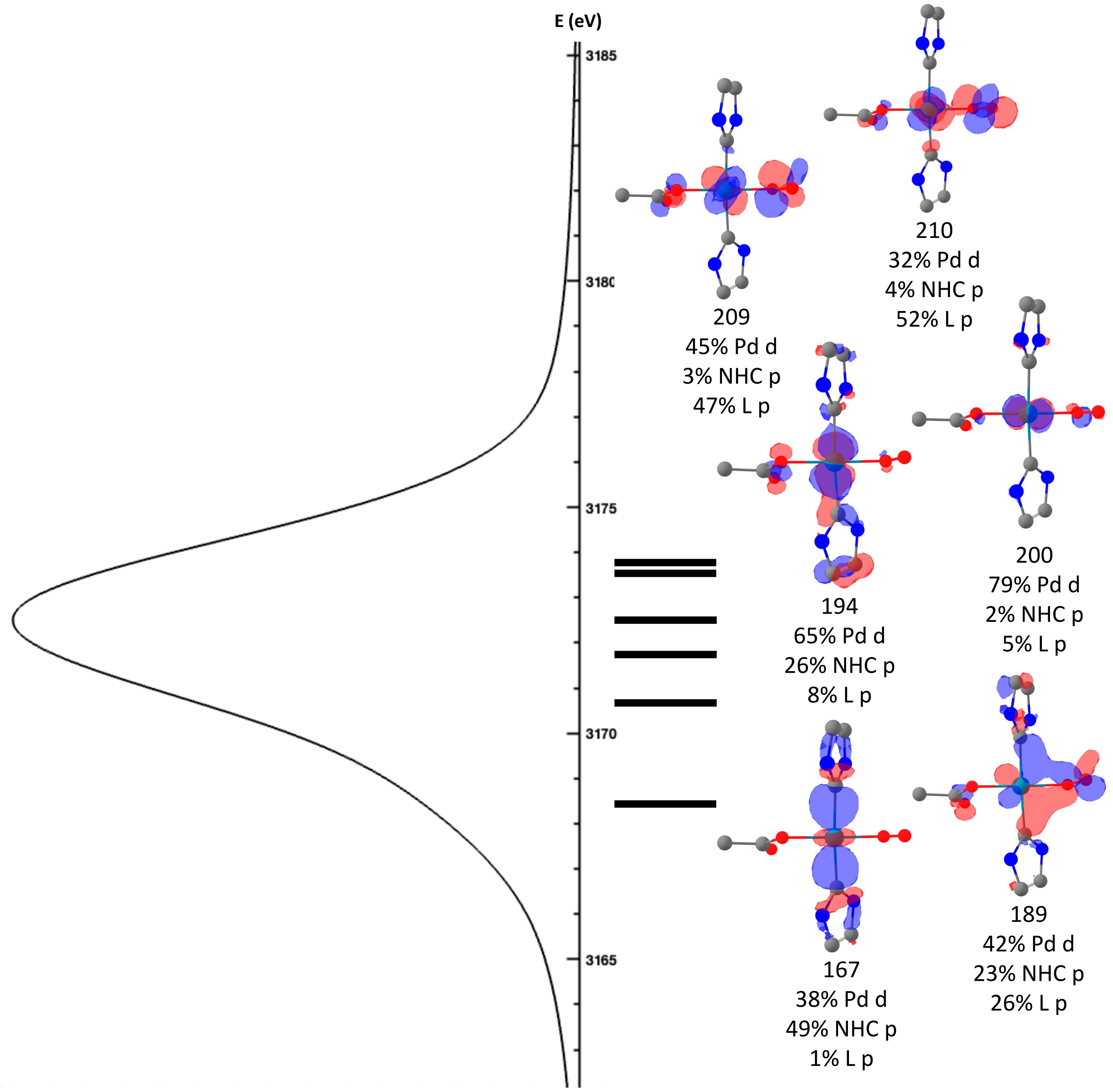

Figure S3: Molecular orbitals corresponding to most intense transitions calculated for 4d-to-2p XES spectra of **3**. A truncated structure of **3** is shown for clarity.

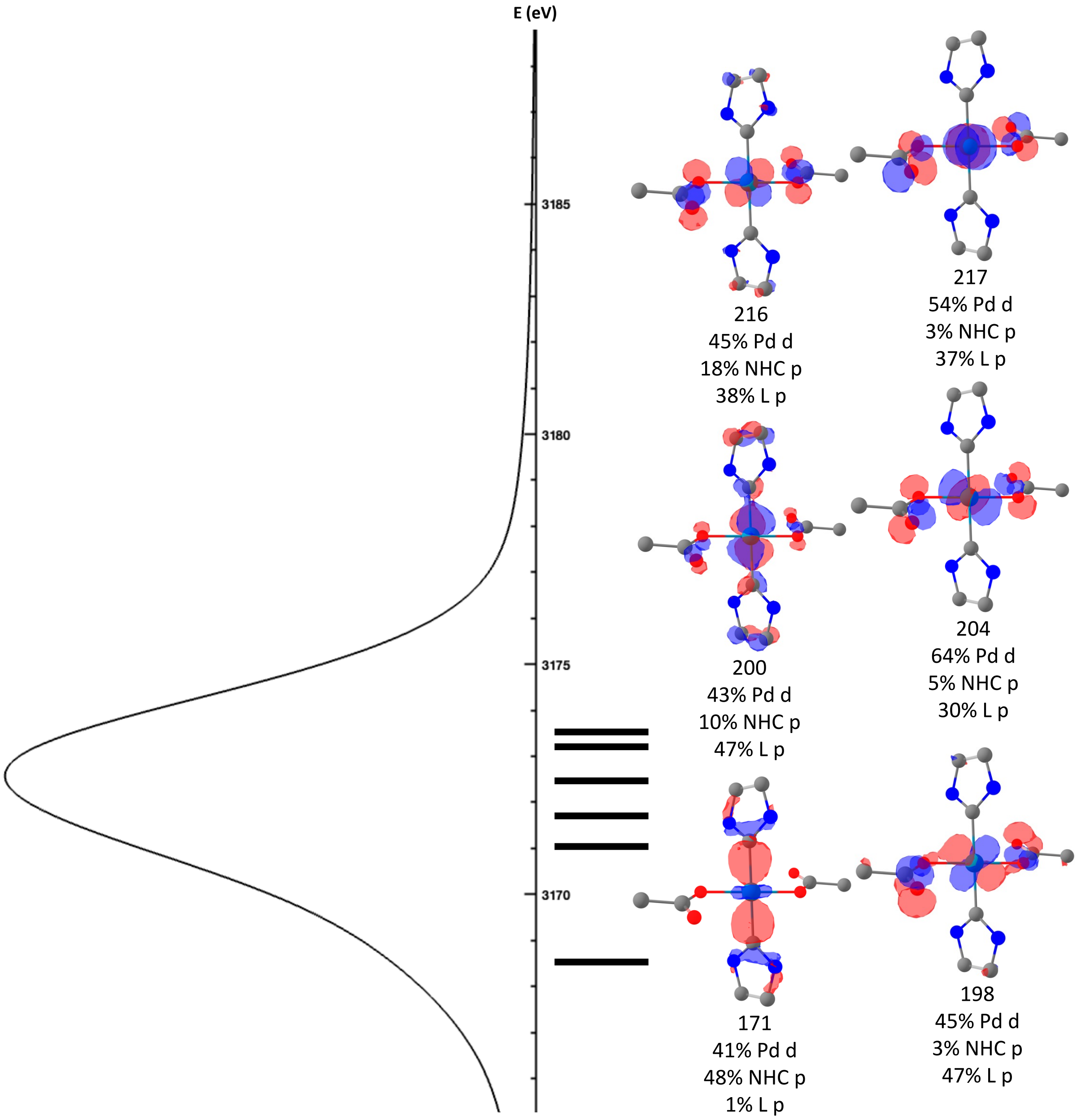

Figure S4: Molecular orbitals corresponding to most intense transitions calculated for 4d-to-2p XES spectra of **4**. A truncated structure of **4** is shown for clarity.

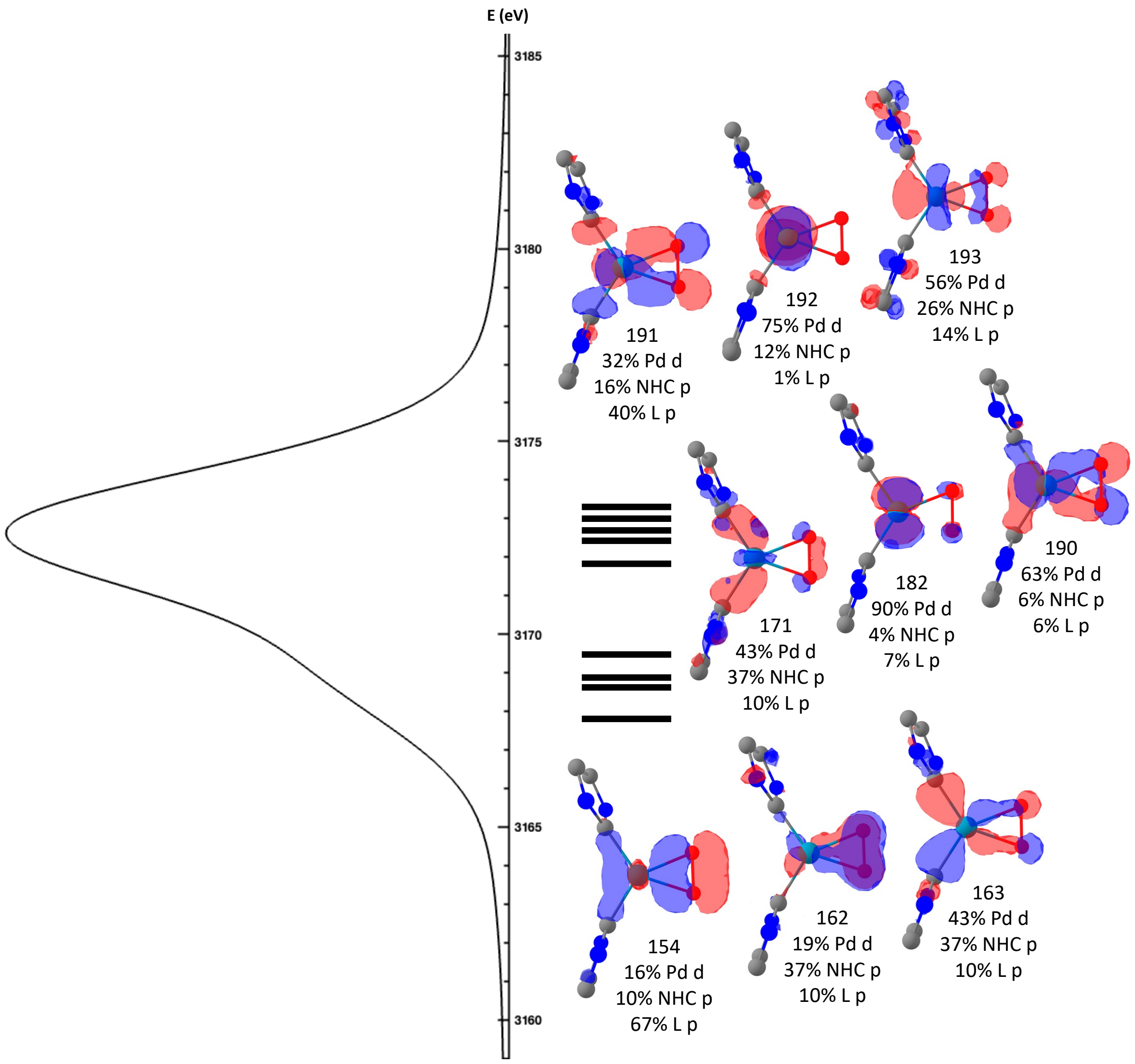

Figure S5: Molecular orbitals corresponding to most intense transitions calculated for 4d-to-2p XES spectra of **5**. A truncated structure of **5** is shown for clarity.

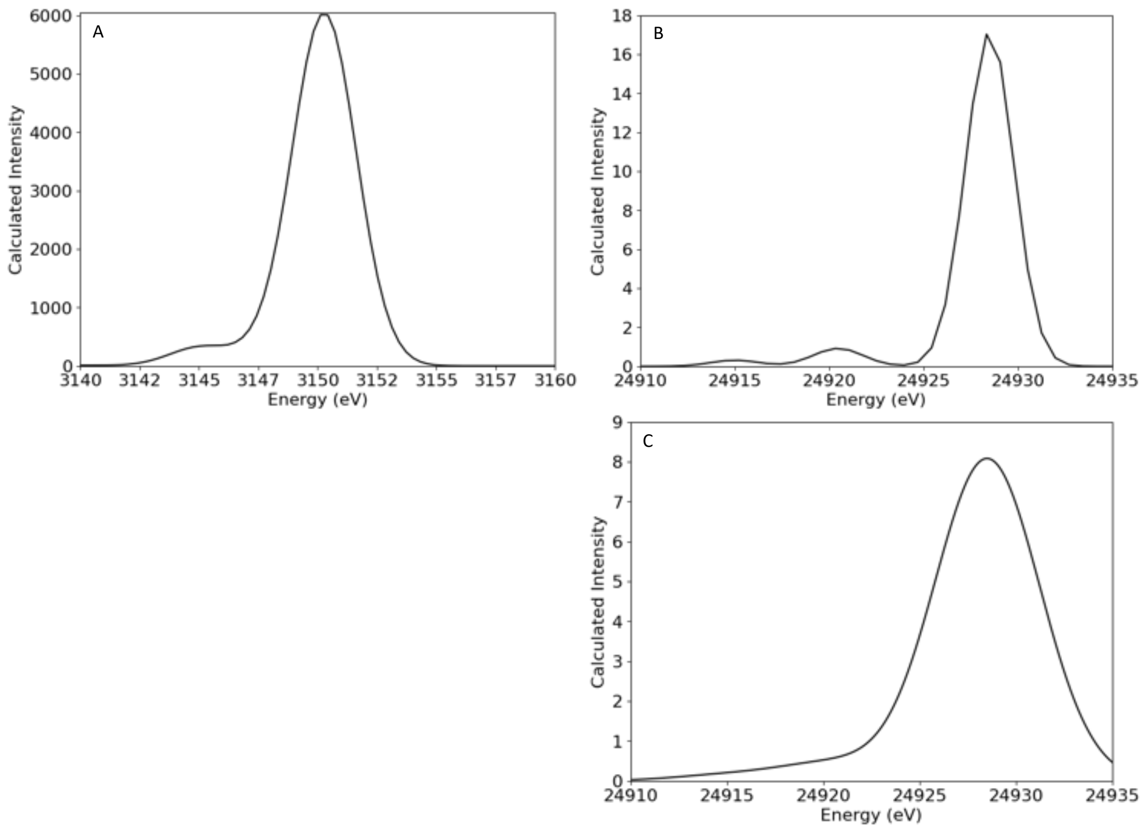

Figure S6: DFT calculated 4d-to-2p XES spectra (A) and 4d-to-1s XES spectra (B) of compound **1** with a Voigt broadening of 2.4 eV (FWHM) applied to each spectra, consistent with the convolution of the spectrometer resolution (1 eV) and the natural broadening of the tabulated L_3 line for Pd (2.25 eV).[2] DFT calculated 4d-to-1s XES spectra of compound **1** with a Voigt broadening of 6.4 eV (FWHM) applied (C), according to the natural broadening of the tabulated K line for Pd (6.24 eV).[2]

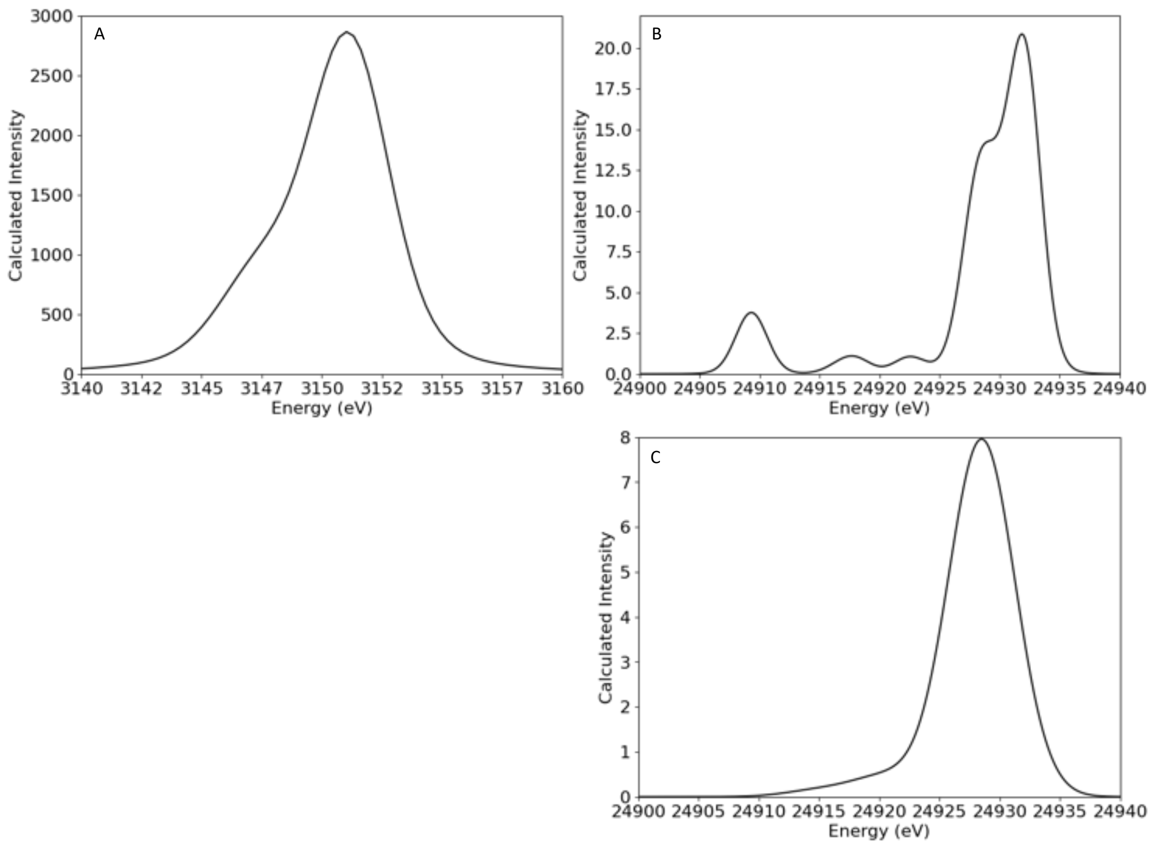

Figure S7: DFT calculated 4d-to-2p XES spectra (A) and 4d-to-1s XES spectra (B) of compound **5** with a Voigt broadening of 2.4 eV (FWHM) applied to each spectra, consistent with the convolution of the spectrometer resolution (1 eV) and the natural broadening of the tabulated L_3 line for Pd (2.25 eV).[2] DFT calculated 4d-to-1s XES spectra of compound **5** with a Voigt broadening of 6.4 eV (FWHM) applied (C), according to the natural broadening of the tabulated K line for Pd (6.24 eV).[2]

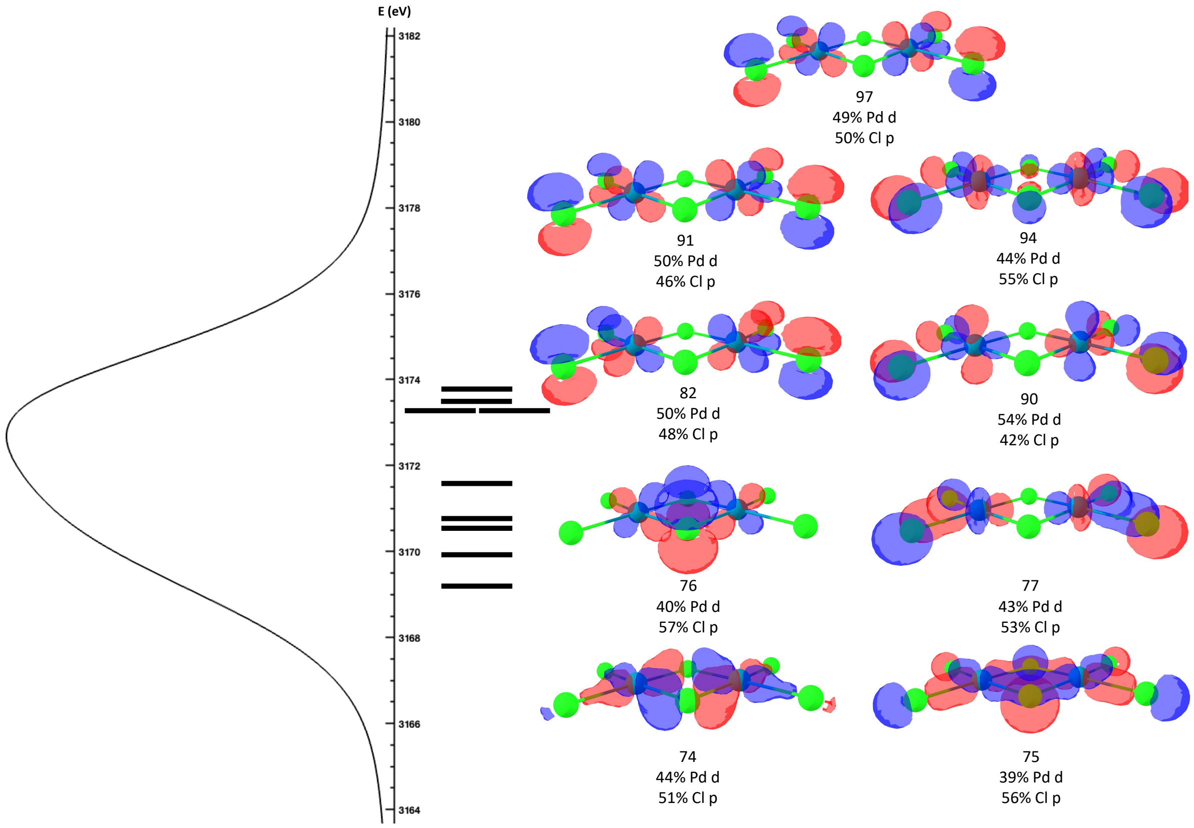

Figure S8: Molecular orbitals corresponding to most intense transitions calculated for 4d-to-2p XES spectra of **8**.

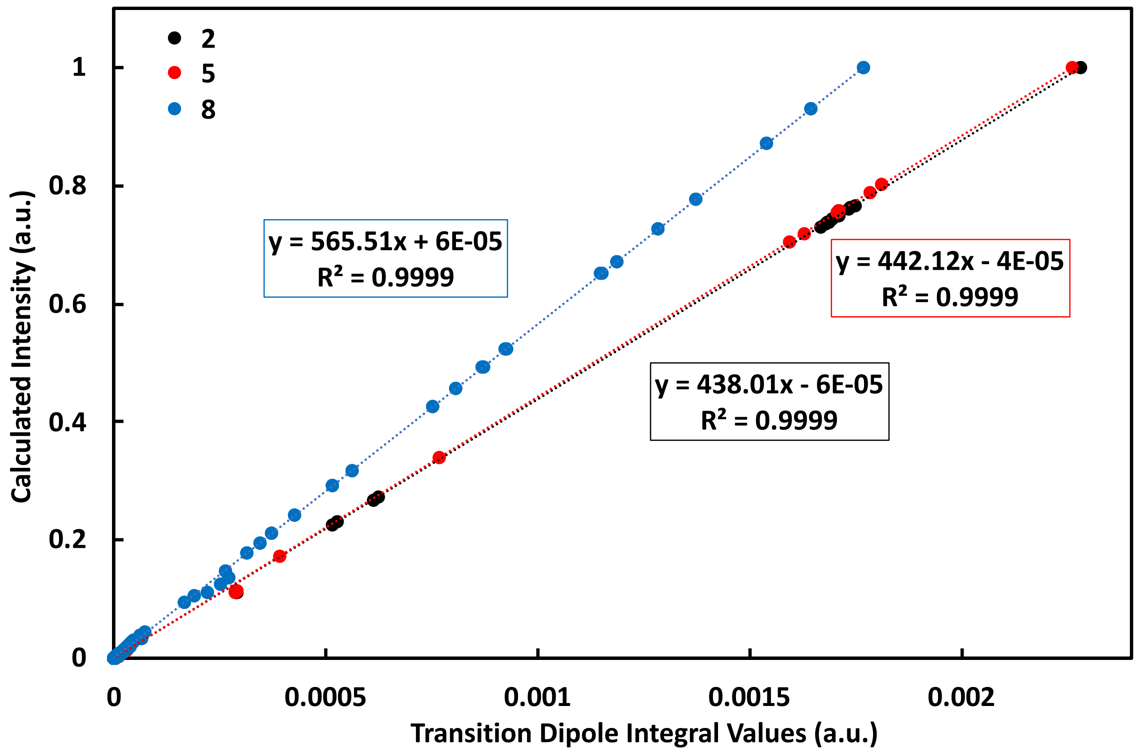

Figure S9: Trend of transition dipole integral values for calculated transitions and intensities of transitions for **2**, **5**, and **8**.

X-ray Photoelectron Spectroscopy (XPS) Measurement Details

X-ray photoelectron spectroscopy was performed on ground powder stuck to carbon tape using a monochromated XR50MF aluminum source (Kα excitation, 1486.6 eV) and a hemispherical analyser (Phoibos 150, R3 NAP HR, 800 μm nozzle diameter) with a 1D-DLD detector. The sample spot size was 1-1.5 mm. The step size used was 0.1 eV and the pass energy was 20 eV. The standard Pd $3d_{(5/2)}$ and Pd $3d_{(3/2)}$ procedure was employed to calibrate the binding energy scale.

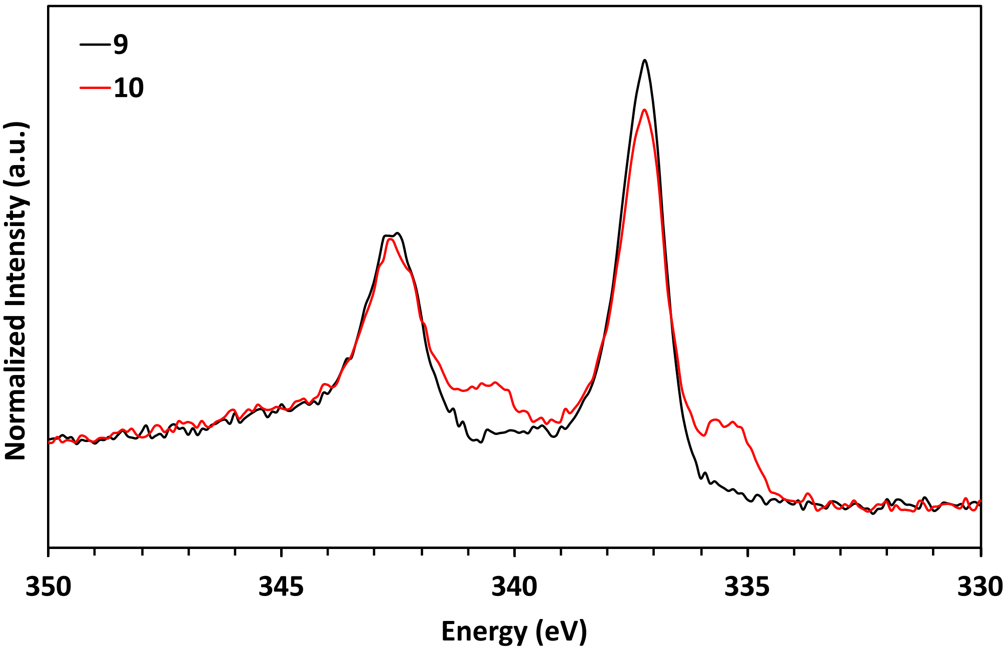

Figure S10: Pd 3d XPS spectra of **9** and **10**.

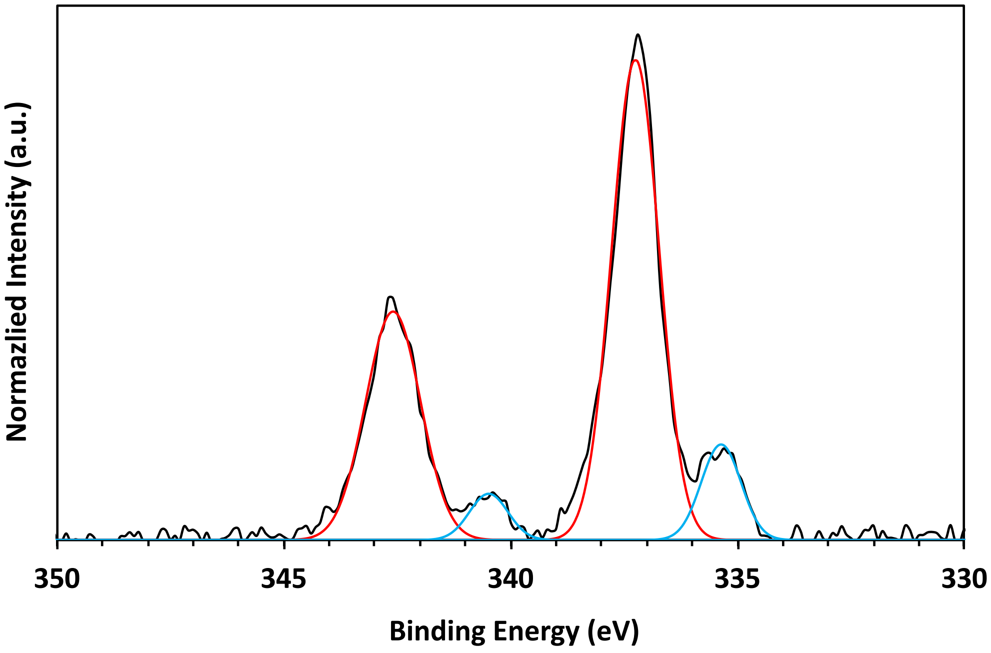

Figure S11: Fitting of Pd 3d XPS spectra of **10**. Relative areas under the red curves is 65% compared to 35% under the blue curves.

Transmission Electron Microscopy (TEM) Measurement Details

Sample was prepared by dry dispersion over Holey carbon film on 300 mesh Cu grid. Analysis was performed using a TFS Spectra – 200 Aberration Corrected Scanning

Transmission Electron Microscope (AC-STEM) operating at 200 kV. The probe current was variable between 20-100 pA. The convergence angle was 29.5 mrad. Particle size was determined using software and methods described previously.[3]

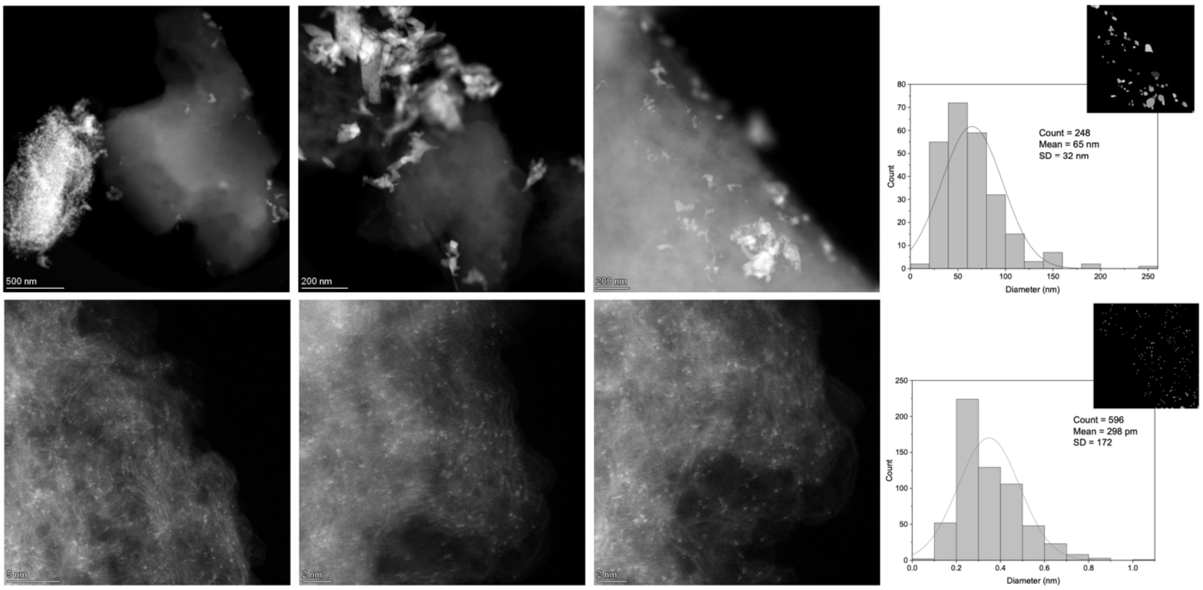

Figure S12: TEM images of **9** and particle size distribution plots.

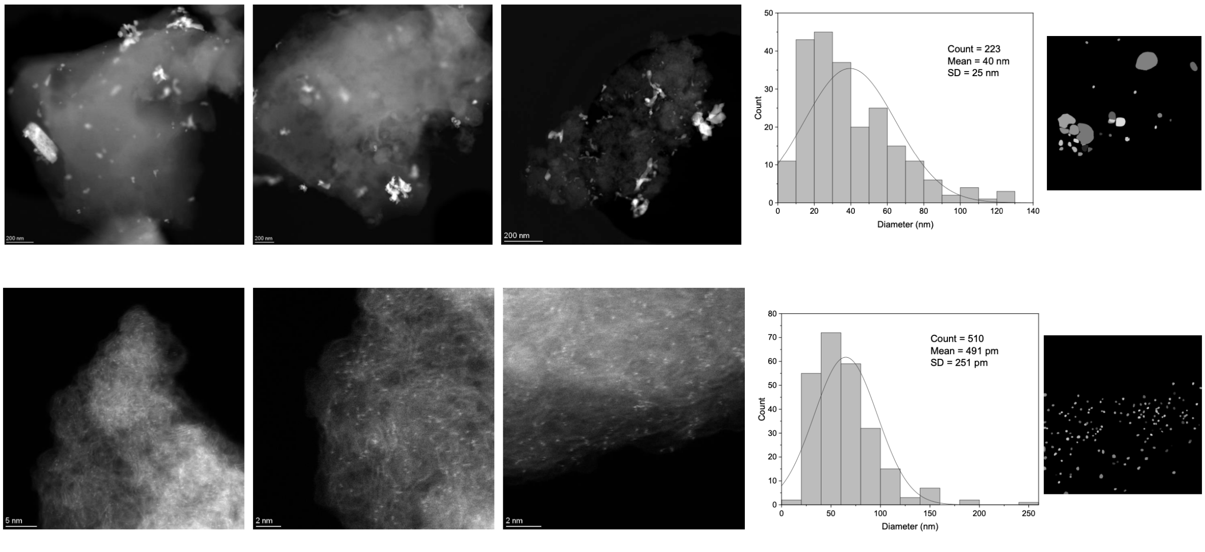

Figure S13: TEM images of **10** and particle size distribution plots.

Input file examples for geometry optimizations and the calculation of 4d-to-2p XES spectra

For more detailed explanation of input files for calculating XES spectra see Ref. 4 and the ORCA manual.[4]

```
#Example input file for geometry
optimization
! UKS PBE0 ZORA ZORA-def2-TZVP AutoAux
Opt TightSCF AnFreq LargePrint CPCM
! UNO

%basis newGTO Pd "SARC-ZORA-TZVP" end
end

%maxcore 6000
%pal
nprocs 8
end


* xyz 0 1

...

*
```

```
#Example input file for XES spectrum
calculation
! UKS RIJCOSX PBE0 ZORA ZORA-def2-TZVP
AutoAux CPCM
! SlowConv TightSCF LargePrint UNO

!MORead
%moinp "PdNHC2.gbw"

%basis newGTO Pd "SARC-ZORA-TZVP" end
end

% xes
    coreorb 2,2,3,3,4,4
    orbop 0,1,0,1,0,1
    CoreOrbSOC 4,5,6,7,8,9
    DoSOC true
    DoQuad true
    end

%maxcore 6000
%pal
nprocs 8
end

* xyz 0 1

 ...

*
```

Optimized xyz coordinates for all complexes

1: PdIPr$_2$

131

Coordinates from ORCA-job PdNHC2

Pd	2.69453806079715	3.43707156771937	3.47506585324891
N	1.39779243661817	1.42193490643233	1.53750044373948
N	3.45754715985034	1.65292002786560	1.05879488471038
N	3.91747517643532	4.95188768757932	5.87539158473190
N	1.97920298416347	5.71113377451110	5.43690287648111
C	2.51112534055797	2.10815522920806	1.93166685634892
C	1.64910690703259	0.57956900260162	0.47499525164713
C	2.95153805783560	0.72609928868368	0.17192925487135
C	0.08515730235191	1.53273953275472	2.09523032092433
C	-0.81893261228549	2.42283773119744	1.50195598127182
C	-2.11792329553572	2.45232952456058	2.00110108670317
C	-2.50429934612644	1.61517695542435	3.03404362489205
C	-1.59452060359158	0.73406785459352	3.59287529022122
C	-0.28105159216077	0.67215761185751	3.13683784450995
C	-0.43862710007106	3.28221927663280	0.31616275461125
C	-1.00254673946508	2.69046914429266	-0.97634517685814
C	-0.88088881661019	4.73224826052003	0.47272176607753
C	0.68004217908945	-0.33632294847276	3.72647994920226
C	0.35092713935932	-1.74492967218318	3.23210869595531
C	0.70479266318584	-0.29342797436478	5.25062099080924
C	4.83484043774283	2.03533892027822	1.01078912640320
C	5.76904046675056	1.26126568222798	1.70910689143120
C	7.11352216543983	1.59925535352755	1.58061840603281
C	7.51112369241380	2.64928421531686	0.77088021979625
C	6.56846394553243	3.38431471303097	0.07261258913002
C	5.21110607920003	3.09370511349941	0.17507412189589
C	5.36864048569707	0.05244060014181	2.52646291844967
C	5.79028317953278	-1.23639273863540	1.82034614666972
C	5.92991846886986	0.10072932885786	3.94253821363987
C	4.20777425380029	3.87257232427878	-0.64690486237225
C	4.31952797035555	3.49619170337590	-2.12433770989053
C	4.35304273622671	5.37934025873727	-0.46078280413845
C	2.87233372573075	4.76631385045039	5.01624543413631
C	3.67975831351379	5.96419147453358	6.78087204208303
C	2.45477985022774	6.44532011476148	6.50249092788545
C	5.15299121724261	4.23117725698197	5.89389870710899
C	5.26056233399615	3.10129062816174	6.71320538066565
C	6.50354124612252	2.48034040603760	6.79614246791821
C	7.59291482472519	2.96573641114905	6.09353304751620
C	7.46129659912580	4.08950782320760	5.29559565957871
C	6.24258328248048	4.75312891684853	5.18589456107986
C	4.09980867320148	2.58264323524142	7.53381668885664
C	3.84862304274534	1.09559522833965	7.30407513508630
C	4.31864203792887	2.86917091068831	9.01931058504721
C	6.14368266915813	6.02775726959904	4.37693399317356

C	6.70335126876777	5.86879823816977	2.96798050276081
C	6.83710634856739	7.17956899854020	5.10518012568512
C	0.68514302927102	5.97219383547770	4.88569158913966
C	0.57097019203716	6.90485097919022	3.84806379848978
C	-0.70787944290131	7.20705828261477	3.38913727877750
C	-1.82583824566675	6.61015361663281	3.94560166103270
C	-1.68657094317995	5.70337537509276	4.98266396332177
C	-0.43032525625594	5.37078370180427	5.48166522566624
C	1.76967503914756	7.61924194502680	3.26438411827882
C	1.83345793565511	7.49160407965229	1.74590074385218
C	1.78017559545807	9.08794138240123	3.68762262450203
C	-0.30758063471958	4.44535244378040	6.67253297870778
C	-1.11963097624752	3.16565169589386	6.50886315917757
C	-0.71395018408457	5.17207450297007	7.95569968885929
H	-2.84187520862224	3.13010704717627	1.56319727284030
H	-3.52366390147686	1.64638418874204	3.40341225321821
H	-1.91097813592707	0.07763050742591	4.39550557272826
H	0.65093224831481	3.27968586725920	0.23723801392128
H	-2.09611540650594	2.69090926799596	-0.95371403345983
H	-0.66824529188711	1.66208555306686	-1.12894004416833
H	-0.68092031598990	3.28503529913436	-1.83559096444270
H	-0.49717652391517	5.17128472920738	1.39536547624031
H	-1.97018202623709	4.82328087148695	0.48090323295193
H	-0.50898503465823	5.32482988888094	-0.36703499589814
H	1.68412556701836	-0.08691469552375	3.37618954425503
H	0.38092265987892	-1.80396992946462	2.14160072846121
H	-0.64774803437246	-2.04747607743908	3.55994846583935
H	1.07033907326440	-2.46414858843699	3.63254116613880
H	-0.25565343528349	-0.59227591322237	5.67818364843294
H	0.94289100261705	0.70789931193490	5.61599002092712
H	1.46353858004302	-0.98278107589636	5.62935225982245
H	7.86083599757248	1.01918400364070	2.11043872617344
H	8.56410756770201	2.89199021429686	0.67688468430144
H	6.89252048995549	4.19540407784778	-0.56989602389656
H	4.27940767425621	0.04627963826019	2.60760308791992
H	5.45289953983832	-2.10625948502420	2.39005612481908
H	6.87903997648942	-1.29207530443363	1.73238555053330
H	5.36668907658294	-1.30084843465756	0.81535636276985
H	5.61123447221497	1.00485295173268	4.46465057500143
H	7.02271673161794	0.07593646252286	3.94403672588425
H	5.57905899149316	-0.76305960447300	4.51315575784809
H	3.20708064769618	3.59709605683089	-0.30658887379022
H	5.30432584807966	3.76374222585426	-2.51763869956059
H	3.56547176953584	4.02920185685066	-2.70944846837200
H	4.17453292868107	2.42392868000537	-2.27509188529917
H	3.57608413086939	5.90143574548371	-1.02499359886134
H	5.32082032348468	5.73791041154071	-0.82061599758110
H	4.25547872680419	5.65973311708351	0.59014238227794
H	6.62072354421204	1.60651552399523	7.42728153543351
H	8.55355935031920	2.46844738450454	6.17297363977474

H	8.32456701496625	4.46965595186375	4.76102557257186
H	3.20081246626888	3.11759417822601	7.21954379081523
H	4.69692197372622	0.48945607184385	7.63229741653558
H	3.66567695707008	0.88337997296526	6.24857602396438
H	2.97195274038106	0.77444779332791	7.87231799688033
H	4.45906712024093	3.93685496838812	9.20404312855522
H	5.20218041891893	2.34170955841791	9.38972557690138
H	3.45637100056025	2.53226376388940	9.60072643951367
H	5.08584299356934	6.28247514622169	4.28090633804135
H	6.21883244391526	5.04754638077132	2.43650388789505
H	7.77915836940638	5.67538747222277	2.98103454362559
H	6.54043261573380	6.78694939948944	2.39757171577082
H	7.90607470961492	6.97760833702053	5.21782845078426
H	6.41608278597988	7.33705455957620	6.10083040419113
H	6.72655245810536	8.10703662366876	4.53706598576646
H	-0.82916032022861	7.92742039325966	2.58783513809812
H	-2.81403063673644	6.85998900438889	3.57467875409176
H	-2.57061555775801	5.25569216478317	5.42231611301935
H	2.66997290297231	7.15081595913853	3.66771316795862
H	0.97566253035858	7.96897790661378	1.26521670350682
H	1.85699076444751	6.44344175203209	1.43933383456174
H	2.73740981895139	7.97659916602984	1.36880424685234
H	1.77911703123825	9.18908842128907	4.77554843971346
H	0.90308653841269	9.61189661676369	3.29758902818920
H	2.67168796657977	9.58679607373007	3.29859384796847
H	0.74225492836851	4.15813678699907	6.76766111806997
H	-0.85926840807539	2.63989060510332	5.58850584377421
H	-2.19330486714285	3.36985158087766	6.49002400847761
H	-0.92717349573751	2.49552976024086	7.35061900639009
H	-1.76604883553613	5.46787662554798	7.91167850214610
H	-0.11639070893442	6.07210190658210	8.11627729691579
H	-0.58349817968506	4.51601115874131	8.82036524972561
H	0.88387101123460	-0.03933824746106	0.03784615137716
H	3.56166575397367	0.26304783165916	-0.58514994487876
H	1.88239127045537	7.23610025558704	6.95723237197740
H	4.39999009567704	6.24457746671128	7.53091914753294

2: $PdIPr_2(\eta_1\text{-}O_2)_2$

135

Coordinates from ORCA-job PdIPr2O22_PBE0

Pd	8.81640473314715	3.93014132965586	7.61277701873762
O	7.24110841651013	5.17908340972555	7.65851570874057
O	7.22060537028754	6.10154749214193	6.75246077074536
O	10.39336962721486	2.68472979540932	7.70321412711353
O	10.45753304120293	1.77767876868289	6.78335911411097
C	7.50441399191313	2.30907368792742	7.56648151499540
N	7.31708772659149	1.37524937454872	8.53284826776499
C	6.42828024452785	0.40021022076654	8.12930581757035
H	6.14522637311809	-0.41700477407058	8.77025636691720
C	6.04479870728050	0.72217633832034	6.88308608561216

H	5.37092222295137	0.23430510459644	6.20015573506176
N	6.70164863679690	1.89067494542108	6.55477709332219
C	7.82116290122363	1.37395779014538	9.87751511256737
C	8.85264354627482	0.48877006475200	10.21461252989315
C	9.22195957776702	0.41390301619632	11.55513416054281
H	10.01357984508566	-0.26548649994418	11.84916004882727
C	8.59360305307120	1.18451731301802	12.51628371563179
H	8.89875409220930	1.10990396608248	13.55440404119344
C	7.57038741913251	2.04479190188704	12.15696917214080
H	7.07134787379366	2.62865594960128	12.92135956881913
C	7.14847165538383	2.14660179977821	10.83488760171257
C	9.52657264826531	-0.41441282840522	9.20446473425777
H	9.24977795776264	-0.07110960323987	8.20587482277407
C	11.04856051823382	-0.35383560056947	9.29911819644688
H	11.41124095888356	0.66769877199735	9.17944014537218
H	11.49065651556884	-0.96629477934095	8.50918194064187
H	11.40733199612361	-0.74205202864475	10.25566792422861
C	9.04717130571368	-1.85774390485290	9.36734116345442
H	7.96356555016657	-1.94270744846101	9.26373459152484
H	9.32278539076839	-2.24517973701225	10.35225462259371
H	9.51245014103411	-2.49620160842928	8.61174436968831
C	5.93579156049513	2.98401487273758	10.49042847334725
H	5.95194489618515	3.17953170293437	9.41653964743749
C	5.92320748668357	4.33571497872879	11.19190321768698
H	6.84112684421438	4.89499493624801	11.00432059916529
H	5.80527298724453	4.23155751237875	12.27343315966228
H	5.08169637665386	4.93079873689229	10.82843981775904
C	4.65577909437871	2.20692102542226	10.80611346393881
H	4.62362602742580	1.24956556311549	10.28178392955785
H	3.77880372073457	2.78787690159188	10.50861330210754
H	4.58440233379077	2.00799617243552	11.87923135034875
C	6.45146310072699	2.51575302581021	5.28378992833897
C	5.33743426556898	3.35936562125494	5.16334699244331
C	5.04695775256914	3.86244363362147	3.89835748394106
H	4.19238321347267	4.51618676238689	3.77021095295850
C	5.81617842579682	3.52475512795357	2.79850924459990
H	5.56791502521151	3.92474739345299	1.82134506535331
C	6.89241700862418	2.66756806763319	2.94078886926172
H	7.47470116025998	2.39248402523057	2.06923476826258
C	7.22922607607285	2.13576674974802	4.18269123443467
C	4.40827100341867	3.65628716039862	6.32147334561420
H	4.94401246168332	3.43389119600021	7.24666831237377
C	3.97513916366084	5.11725229257460	6.38572619626705
H	4.83739366658253	5.78461448613853	6.42020845409109
H	3.37692425820458	5.27927447856639	7.28618422213340
H	3.35217199062638	5.39118928129490	5.53052916122500
C	3.17630241515001	2.75035139224489	6.25638875369415
H	3.44447213033332	1.69237288103004	6.25615952011942
H	2.60256531732486	2.95378714960303	5.34759695245945
H	2.52711978294552	2.93746281012859	7.11576958968603

C	8.33909475667758	1.11398655407189	4.29058814412680
H	8.63164542360613	1.04251041129194	5.33926687146009
C	9.58641955331907	1.50921530432014	3.50948503603039
H	10.38575570088500	0.79040799634092	3.70618539597938
H	9.94393361203705	2.49885237354350	3.79895004854153
H	9.40811925633122	1.51254768100430	2.43095876218589
C	7.84122011812295	-0.25802734098026	3.83230704893843
H	8.63192580069410	-1.00399036694719	3.94933848253472
H	7.55548736038077	-0.23032888914562	2.77670010109481
H	6.97397908969032	-0.58920688233939	4.40734416336130
C	10.12396484204898	5.55050250511986	7.65998061595152
N	10.97234356536540	5.99458823143583	6.69808206099805
C	11.60880992167334	7.15632918573035	7.08575227055626
H	12.31224777157438	7.66239522370064	6.44737131006835
C	11.16504987924850	7.44705106633220	8.31981429162320
H	11.41271993905921	8.24916245432554	8.99355633662177
N	10.26112455693788	6.46058310211400	8.65640621430869
C	11.28176276074428	5.40049700979941	5.42516220049417
C	10.55118699741930	5.80125299828718	4.29925038719928
C	10.94366249526758	5.29643782058288	3.06239193202369
H	10.39932117774787	5.58877094298407	2.17213634588426
C	12.02756373091647	4.44476539297364	2.94895144678317
H	12.31920471331607	4.06593292158790	1.97536010247942
C	12.75013852312971	4.08723350144134	4.07378731575449
H	13.61265083228110	3.43974878464983	3.96917903930491
C	12.40403510901746	4.56411875651600	5.33483520414760
C	9.43338046221578	6.81711860053485	4.37794878772647
H	9.09829923944343	6.87203678288496	5.41480397937433
C	9.94354589945775	8.19733426685243	3.96035650065297
H	10.78442192490282	8.52335048558189	4.57628429267410
H	9.14544294886717	8.93862400864043	4.05490625020005
H	10.27344766445127	8.18629333431833	2.91740432336589
C	8.22043914296955	6.42633285429523	3.54240151451364
H	7.85394937274720	5.43418775510476	3.81069539907621
H	8.44400769641610	6.43019668324018	2.47239298069380
H	7.41203176523265	7.14232580368207	3.71019253621842
C	13.28801358340240	4.25020464406995	6.52336215720228
H	12.71777532640485	4.45877191303579	7.43093016591870
C	14.52049181361111	5.15806359256629	6.51662329017684
H	14.25106682273866	6.21576774635000	6.51582267411076
H	15.12989926501774	4.96343631316262	5.62950962787491
H	15.13520446273063	4.96346287977234	7.39938875043519
C	13.72066317322102	2.78891542321463	6.58252323472700
H	12.85924648322364	2.11967425886146	6.57253639022061
H	14.28376363452234	2.61375895417743	7.50291988785357
H	14.37701959894895	2.52906962463076	5.74811896160477
C	9.68510679143345	6.42837933666100	9.97160688600757
C	10.29717123376980	5.62225918106405	10.94217121912115
C	9.80142720395136	5.68859414037811	12.24062700632751
H	10.25163435790902	5.07818301042919	13.01441885177676

C	8.76433292092530	6.54541614726317	12.56697529492887
H	8.40088733215731	6.59145901589214	13.58784825697627
C	8.19556868307431	7.34784373281539	11.59496210832475
H	7.39116240284737	8.02290487137492	11.86298828913877
C	8.64014978130609	7.30965329213222	10.27594139216905
C	11.52039879408582	4.78327048158383	10.64094898337006
H	11.55493388725266	4.60865233722960	9.56392272150105
C	12.78778290707353	5.54556197797618	11.03473655544933
H	12.85030153976886	6.51351486496689	10.53316479061076
H	13.67532646509026	4.96554522267635	10.76851224221794
H	12.80721270129231	5.72172803012199	12.11405678094866
C	11.49118235093718	3.41827639592918	11.31547628229359
H	10.58381927329142	2.86553591155743	11.06676804318142
H	11.55032685161166	3.50106302810936	12.40360493217130
H	12.34995919539405	2.82740010694911	10.98730076484406
C	8.02425101088627	8.24341670247695	9.25639760542752
H	8.35139245175686	7.92545355142466	8.26469228509463
C	8.49984733793929	9.67968230345961	9.48053272144879
H	9.58718423886941	9.76327484872312	9.42716786146993
H	8.18242179653192	10.04233245721081	10.46236950697399
H	8.07048089697007	10.34027216892698	8.72266990716787
C	6.49911332497802	8.18830651392501	9.27050847974536
H	6.13940206280722	7.17063496009163	9.11388809995426
H	6.10087229166780	8.81695733177024	8.47008003782403
H	6.09198056235702	8.56030724792159	10.21409716918663

3: $PdIMes_2(AcO)_2$

109

Coordinates from ORCA-job PdIMes2AcO2

Pd	0.00059543807342	-0.00060838669193	0.00055399012057
O	-0.74022527680055	-1.56991985329238	1.04395158457861
O	1.22302306145822	-2.51433249874300	1.56129601076490
N	-0.50871587270623	1.14504848477045	2.83205328214396
N	1.45360268727972	1.67954379221837	2.18259102931924
C	-0.00897853037306	-2.47211473989879	1.58393485115682
C	-0.77594295781880	-3.57294072325604	2.28473414019590
H	-0.68767077336455	-4.48735562528032	1.69407468257408
H	-1.82990081511538	-3.33062205854932	2.40622306909448
H	-0.32364530813568	-3.76747617971817	3.25756094941793
C	0.33536302505740	1.02413152533665	1.77537727891065
C	0.07192799559721	1.84884096457157	3.86693928079378
H	-0.45302350566216	2.04616023301135	4.78618783575630
C	1.30709471208966	2.18249998362644	3.45917131916845
H	2.09435204581062	2.73240805788716	3.94657292594146
C	-1.86196915326596	0.68932292436765	2.94918128718246
C	-2.13606831878876	-0.37644366050155	3.80595652251161
C	-3.46255571052688	-0.76511108968040	3.95981493849173
H	-3.68658447348129	-1.60425877974582	4.61159827349416
C	-4.50149718804977	-0.10473790726425	3.31497412816865
C	-4.18813064657682	0.97959477095439	2.50294308165178

H	-4.98733851789987	1.52701949950997	2.01215525194653
C	-2.87762143969280	1.40489757739045	2.31367979945599
C	-1.05817573378560	-1.06189682280860	4.58750845105793
H	-0.14837005051529	-1.19923756604090	4.00213764615461
H	-1.40103070503051	-2.03580460977323	4.93644210345967
H	-0.78579306867889	-0.47247349470783	5.46860665666102
C	-5.92688054039863	-0.51406535324730	3.53116443343416
H	-5.99793642084288	-1.56033166929236	3.83262138543596
H	-6.52190446072079	-0.37048738003745	2.62743492875753
H	-6.38498650208333	0.08876256550494	4.32174938318240
C	-2.58975344838575	2.62406332911278	1.49551126002402
H	-2.07245682149536	2.38224337499154	0.56384042898772
H	-1.95223374633790	3.32256016120981	2.04479401167344
H	-3.51973151095498	3.13603286504463	1.24509979755995
C	2.64327726347488	1.97795697921450	1.43899363113777
C	2.76887497580039	3.26285228019098	0.90328902276964
C	3.94813378157944	3.58427408618889	0.24592220211097
H	4.05213749659758	4.57700401606117	-0.18208225519380
C	4.99631344415856	2.67518058749233	0.13195192834097
C	4.84661566493519	1.42365297751462	0.71402971576720
H	5.66449764938212	0.71132910205161	0.66290457151458
C	3.68400133309622	1.05482875827559	1.38914284513442
C	1.68968337105398	4.28793398559154	1.06473749482613
H	1.87805761958010	5.14497681728546	0.41841001115661
H	1.64698846655516	4.65040000479194	2.09612073890150
H	0.70485665692496	3.88112865545129	0.82831927503114
C	6.25908361055864	3.05522459449227	-0.58006753327332
H	6.99728964206611	2.25387123700384	-0.53179342002359
H	6.69957614073086	3.95489178549759	-0.14202322713055
H	6.06420778366753	3.27397935569910	-1.63350867909144
C	3.60263927971598	-0.26470330634199	2.08915850995541
H	3.53877327538568	-0.11917463436159	3.17251101819896
H	4.49562119676755	-0.85656028838141	1.88436572090032
H	2.72610128404693	-0.84377038909858	1.79264512729116
O	0.74116278115376	1.56880657612157	-1.04283195941381
O	-1.22229680498549	2.51287401839527	-1.55996290589803
N	0.50903827696873	-1.14524485025745	-2.83134537536583
N	-1.45303581851495	-1.68020684369000	-2.18152890825288
C	0.00972115775535	2.47096913389916	-1.58259349352525
C	0.77644806086986	3.57170129499280	-2.28379535679039
H	0.68915458742524	4.48592731104429	-1.69269557494251
H	1.83020530220877	3.32900595882491	-2.40634574242951
H	0.32330903798744	3.76676504062237	-3.25612302308537
C	-0.33473092195025	-1.02489168057762	-1.77434329767698
C	-0.07161882618903	-1.84916295051539	-3.86613041382148
H	0.45317085934596	-2.04622335754671	-4.78552871413316
C	-1.30665822380787	-2.18304741471236	-3.45817365548837
H	-2.09394211703400	-2.73297238326970	-3.94551201572976
C	1.86200627450403	-0.68882030798212	-2.94904967809957
C	2.13521976454200	0.37681042320777	-3.80623439762948

C	3.46147088402687	0.76616850362225	-3.96067317129799
H	3.68482155951805	1.60520406779112	-4.61282825893196
C	4.50098243628420	0.10658034979775	-3.31600479051749
C	4.18848126212426	-0.97770431573190	-2.50352733789472
H	4.98815176755849	-1.52456568760381	-2.01285902145785
C	2.87829966090249	-1.40368251069476	-2.31371732706593
C	1.05672890976822	1.06130535625287	-4.58779206249834
H	0.14686529901653	1.19796837420564	-4.00236809715367
H	1.39876751163891	2.03546152390437	-4.93682740567664
H	0.78474880835039	0.47158391103899	-5.46881914156140
C	5.92611164811926	0.51658634322401	-3.53263837269694
H	5.99608581402522	1.56100875677809	-3.84062821357348
H	6.51988314661475	0.37968613038226	-2.62702812606321
H	6.38638674955119	-0.09069545183754	-4.31852791410708
C	2.59136437606137	-2.62267712319383	-1.49497888048762
H	2.07449143010065	-2.38069542429419	-0.56311729106274
H	1.95381094530928	-3.32163127724942	-2.04363516213155
H	3.52167945699256	-3.13418079160347	-1.24487147388553
C	-2.64285325883313	-1.97840773760768	-1.43807593390113
C	-2.76887918979267	-3.26337294175566	-0.90263964478966
C	-3.94858699014390	-3.58482396159441	-0.24608899220142
H	-4.05295643815806	-4.57762576250630	0.18165916753083
C	-4.99683169738246	-2.67572149306766	-0.13278829251347
C	-4.84665543778538	-1.42409238323154	-0.71452642320935
H	-5.66455763859291	-0.71175688600567	-0.66390744599914
C	-3.68355536101762	-1.05521585634796	-1.38876768163509
C	-1.68977896589831	-4.28856547667123	-1.06391713487714
H	-1.87850549426468	-5.14576761617099	-0.41790554038645
H	-1.64675587892632	-4.65072226809763	-2.09539516243826
H	-0.70498675581223	-3.88195183553869	-0.82703550464921
C	-6.26028755617309	-3.05592595605982	0.57793021195331
H	-6.99857287937598	-2.25469593662738	0.52879293630638
H	-6.70017891704819	-3.95564957639307	0.13939563094177
H	-6.06657160564447	-3.27464410972020	1.63158650079220
C	-3.60175855816567	0.26433638575230	-2.08869493933288
H	-3.53804093756228	0.11884294518380	-3.17206560626419
H	-4.49451317683693	0.85649906702454	-1.88380112283350
H	-2.72500572278367	0.84307238049243	-1.79217115969964

4: PdIMes$_2$(AcO)(OOH)

105

Coordinates from ORCA-job PdIMes2AcOOOH

Pd	-0.03198950707891	-0.00713445397078	0.02574361895900
O	-0.25607066652809	-1.77432432875221	1.04415166868437
O	1.87370328001774	-2.13865846559066	1.63755279315478
N	-0.39789866472633	1.00355081978882	2.90109704824447
N	1.29979037554633	1.95704429740153	2.02567197371828
C	0.67884060581688	-2.44396293172686	1.60376825184749
C	0.21542762716375	-3.74288947447976	2.23119592681492
H	0.21150430584413	-4.51662793514534	1.45756303204336

H	-0.79752649388752	-3.65850546122309	2.62410186707593
H	0.90152634781128	-4.05311426798000	3.01840686442924
C	0.33848977845245	1.03784198588673	1.76306524933165
C	0.09451065639018	1.87894276171286	3.84859680523303
H	-0.36591093475120	1.99252625477855	4.81536344449228
C	1.16439923468035	2.47740721168407	3.29736104274054
H	1.84108431622703	3.22137792397471	3.68290164299175
C	-1.57340350120129	0.22579896475917	3.14917819958363
C	-1.50075014819770	-0.82359818146283	4.06580730516794
C	-2.66381532483242	-1.53538890349456	4.33670689720349
H	-2.61668074327450	-2.36001555896706	5.04166793328438
C	-3.87625538503166	-1.21875400573000	3.73484971431720
C	-3.91157765388657	-0.14592085410078	2.85150164189319
H	-4.85446764091443	0.13459860195534	2.39224442019145
C	-2.77555962112542	0.59634439028342	2.54581712780986
C	-0.22307457480738	-1.18019017637768	4.76224939729314
H	0.62808366790418	-1.16577681498315	4.07944653148785
H	-0.29794366287998	-2.17365747756574	5.20489321763869
H	-0.00157652084298	-0.47324228091170	5.56725066106230
C	-5.10831994892211	-2.02292143098387	4.01954193355600
H	-5.19116588209801	-2.86291719509694	3.32209945180084
H	-6.01065312108169	-1.41871202877222	3.90975089138747
H	-5.08636542523750	-2.43832199337746	5.02867464925018
C	-2.86512789870291	1.76655606851744	1.61596347958572
H	-2.33948101722317	1.57238687640469	0.67705690222958
H	-2.41114318634298	2.65875404959810	2.05563346952370
H	-3.90783951223998	1.98808168130319	1.38663855332353
C	2.33569123265530	2.42609428691535	1.15205695809839
C	2.13981325178960	3.63186774084369	0.47568104177422
C	3.17562483699711	4.10848987885731	-0.31968275837414
H	3.03269206375939	5.04079520479959	-0.85787901072772
C	4.38528113854180	3.43190566055010	-0.43528893699362
C	4.55739078718088	2.26209180173997	0.29479162922176
H	5.50738249851923	1.73813488405364	0.24700163384899
C	3.55148301412331	1.74407616781361	1.10535234993988
C	0.86902078352125	4.40938644475147	0.61360659767946
H	0.86708793637332	5.26135713746370	-0.06706319578750
H	0.74653458101658	4.79145760911220	1.63132806059093
H	0.01066096124088	3.77490667340103	0.38812689562247
C	5.47216847777632	3.94657220811907	-1.32939017174768
H	6.45904310839254	3.67081844746670	-0.95304186464366
H	5.42595854043906	5.03278742455858	-1.42609194560608
H	5.37645905549151	3.52400131680572	-2.33486469029903
C	3.80245182139716	0.52332602770279	1.93344557565218
H	3.72921489847667	0.75743501025951	3.00028074673737
H	4.80522877232811	0.13914371107620	1.74253971035354
H	3.08005333526056	-0.26962020030847	1.72893699001218
O	0.14312647601638	1.72011893027775	-0.93566349464946
H	-1.04483323141633	1.71647889636947	-2.41428402700928
N	0.51106450674059	-1.27032824112328	-2.72544969727406

N	-1.58736481460760	-1.35518770958630	-2.34725780544081
O	-1.09153028118865	2.11977529112208	-1.53785343179615
C	-0.41497424954929	-0.96550625797121	-1.78476748695918
C	-0.06565893644297	-1.83854106355500	-3.84272312632165
H	0.51868060498915	-2.15022971868773	-4.69222645210830
C	-1.38837534664932	-1.89299634773583	-3.60427874895123
H	-2.20518671102979	-2.26077238652812	-4.20238481243970
C	1.93139514914647	-1.13898066317077	-2.59124690920706
C	2.56759811658379	-0.00326682927815	-3.09261511685195
C	3.95248614857009	0.06506589408615	-2.98307865860936
H	4.46206927301778	0.94391996546670	-3.36659064729266
C	4.69714097257710	-0.95654664912251	-2.40394835273898
C	4.02704799409095	-2.08894136862489	-1.95392440221128
H	4.59379122293319	-2.91062034854224	-1.52640972641236
C	2.64498796219353	-2.20903181937908	-2.04925105617334
C	1.79755307656790	1.10857105204438	-3.73173162576598
H	1.16039723077945	1.59475798405036	-2.98758274419754
H	2.47695817370414	1.85171154230478	-4.15003681025698
H	1.15299158197294	0.74141925177597	-4.53428255406983
C	6.18341516720984	-0.83420629568159	-2.25952643104771
H	6.43990475402974	-0.37153320218847	-1.30086477349461
H	6.66743862299950	-1.81200299015205	-2.28953433161900
H	6.60798021153044	-0.20860653540062	-3.04671339532289
C	1.95742700029489	-3.46971945786431	-1.62616789376988
H	1.10021876454369	-3.26369804522067	-0.98364876531501
H	1.58307283044167	-4.01769291881182	-2.49640114194754
H	2.64733400318108	-4.12034782187606	-1.08899809223499
C	-2.89012865447270	-1.33464206403785	-1.74659891845264
C	-3.26057560121875	-2.42343331815689	-0.94674282152670
C	-4.53215389109020	-2.42573393340947	-0.39127288082721
H	-4.82645088561902	-3.26449213218799	0.23230899997598
C	-5.44212312260507	-1.39900196825752	-0.63147145002626
C	-5.06113422074846	-0.37443783773978	-1.48436114451298
H	-5.77313955926988	0.40914287559616	-1.72655259171131
C	-3.79578323539549	-0.32461383630293	-2.06789107715153
C	-2.34011966075219	-3.58514297929582	-0.73952508738536
H	-2.81625955740402	-4.33820628230583	-0.11109552571597
H	-2.08017256081124	-4.05221997084243	-1.69389340504452
H	-1.41180758431261	-3.26741927524376	-0.26224001835514
C	-6.79752831412516	-1.41023453563392	0.00641883121448
H	-7.51149933598395	-0.81848633625725	-0.56869885857604
H	-7.18192616118995	-2.42755872165158	0.10267516507886
H	-6.75181291090557	-0.98434662199748	1.01386670638645
C	-3.49805321719624	0.77567091946940	-3.04102018408039
H	-2.59132855292647	0.59190067003655	-3.61653488402232
H	-4.32629707277337	0.87849849073850	-3.74533650404142
H	-3.39016642975018	1.73317961714521	-2.52606106244046

5: PdIMes$_2$(η_2-O$_2$)

97

Coordinates from ORCA-job PdIMes2O2_PBE0

Pd	0.28324785138892	12.90608446792107	5.06722062841797
O	0.93579994495717	14.00910139897359	6.60669435563927
O	0.18642514567114	14.79380122869744	5.72612974467877
N	2.18282580506589	10.45606154448566	5.13497419712502
N	0.48330349095362	10.14464830261197	6.38461568525482
N	-2.07873786543640	12.72391144648310	3.04723435107975
N	-0.33119987117366	13.55856079507786	2.15485914693954
C	0.98367228351591	11.01059247454850	5.46059050892510
C	2.41184020936549	9.28456837682353	5.83079619837878
H	3.31527519437554	8.71336066626686	5.69985690831730
C	1.34129571147127	9.08798475212604	6.61547476064489
H	1.10627683700527	8.30731213524159	7.31925070200682
C	3.19518407286651	10.95366803458142	4.25419910308852
C	3.38694467100214	10.30362618040509	3.03299534427120
C	4.46990110421214	10.68658203526375	2.25148773695928
H	4.62684290237565	10.18820899963726	1.29943483478478
C	5.35718578791321	11.67603672508544	2.66047395444555
C	5.14128269805422	12.28672434327579	3.88976917315361
H	5.83791722618477	13.04364135494914	4.23742352977484
C	4.07683504692606	11.93356995410302	4.71407027322711
C	2.49139431631374	9.18705709086659	2.58994528499704
H	2.60026589934792	9.01399460256326	1.51921218087824
H	1.44194449593677	9.39636105029643	2.80494011489783
H	2.74480624453229	8.25557598581467	3.10553053162997
C	6.51532453959584	12.06945546116709	1.79535482089461
H	6.17356235998882	12.63290250543652	0.92208702256469
H	7.04626929061439	11.19003321562884	1.42322649523642
H	7.22221472817351	12.69551859302942	2.34149445034020
C	3.93735668055675	12.54840248766333	6.07160182839744
H	3.01828381388747	13.13545770707047	6.16085796434878
H	4.78800822175022	13.19826448012635	6.27978188743282
H	3.89953174282535	11.77744780142490	6.84697118965564
C	-0.73722199455543	10.23843163252330	7.12707381359134
C	-0.75529418376778	10.96845550176228	8.31872747332219
C	-1.92343882641781	10.94886879032260	9.07207134671821
H	-1.95171975803951	11.50991351686253	10.00154700215270
C	-3.03501264648241	10.20380783439237	8.69157607486268
C	-2.95622818092771	9.44993795778847	7.52723579761751
H	-3.80116929491243	8.83742687409580	7.22905222504314
C	-1.81424175148421	9.44510631607785	6.73271138991869
C	0.45009546207557	11.70461602913500	8.81362936131916
H	1.33091414167766	11.05675978919172	8.82416721284512
H	0.27933313961727	12.06416800954531	9.82901215519493
H	0.67790236510151	12.56182919333631	8.17413273785635
C	-4.28091983709837	10.21306766598223	9.52426008959103
H	-4.04364979353187	10.24233774014940	10.58975039546516
H	-4.89542043146600	9.33330982991901	9.32703676907553

H	-4.88820984004813	11.09649738191625	9.30279459396946
C	-1.72779156716894	8.55819523185910	5.52992546225147
H	-1.09354798511373	7.68966918216615	5.73298999192040
H	-1.29433572346684	9.07716139414546	4.67399133353712
H	-2.71644108989412	8.19423942139321	5.25205415583874
C	-0.76152021603599	12.95295682921614	3.29583719680956
C	-2.44747186925722	13.17155475264624	1.79250672083833
H	-3.45702002980486	13.07791470066898	1.42972464184376
C	-1.34646376583994	13.69445448308831	1.23049505921504
H	-1.18491224265175	14.15291110035124	0.26942032084185
C	-3.07674136210919	12.16747113457915	3.90939083148755
C	-3.66085737319372	12.97564702629266	4.88725728097482
C	-4.74029154598436	12.45951309739352	5.59692309122304
H	-5.20846063719678	13.08148337325542	6.35392640630221
C	-5.25579685360704	11.19422201786933	5.33904718235398
C	-4.65627324192145	10.42914490670565	4.34561374346406
H	-5.04981543849041	9.44212301202705	4.12197101601956
C	-3.56979402357743	10.89491314132707	3.61431060732257
C	-3.20165079647273	14.38001815202526	5.12707862163558
H	-3.80408957968810	14.84538544544416	5.90782369137021
H	-3.30089300753256	14.98170336055728	4.21820643339768
H	-2.15005812690317	14.42205439284875	5.42350042660683
C	-6.42320943269878	10.66594366389291	6.11480793373460
H	-7.05915677993551	10.03434300238429	5.49142690302552
H	-7.02864658672080	11.47777778324872	6.52150952304055
H	-6.08297105830992	10.05504593260634	6.95651301681789
C	-2.98896532610914	10.06326938350685	2.51153728508076
H	-3.30026147762746	10.43545935342342	1.53099984033043
H	-3.32643441465010	9.03026509162647	2.59713423742743
H	-1.89731647525877	10.07587054173543	2.52620269721910
C	0.97640522528681	14.06959852859118	1.87807010101981
C	1.27852420044968	15.38852372490260	2.22447655594968
C	2.53962318578604	15.87162563900822	1.89413062553929
H	2.79273766171128	16.89226170460690	2.16524096868690
C	3.46835141025503	15.09748382976882	1.20655137081361
C	3.09851623779218	13.81586616510184	0.81522763833011
H	3.79277989228234	13.21070931020598	0.24150344225307
C	1.85419542287656	13.28328973014361	1.13336784733989
C	0.27168988332058	16.27980157590446	2.88186151107726
H	-0.65085637108776	16.32854278589122	2.29698401970635
H	0.66893986139337	17.29092069684893	2.97699598505327
H	0.01736526189122	15.91220275733989	3.87823723730821
C	4.83305752245976	15.63455985229966	0.90059024023070
H	4.81389330180681	16.71910754687264	0.77862957020420
H	5.24347999070365	15.18692735445811	-0.00628385332290
H	5.52610968225864	15.41007963702266	1.71793281601862
C	1.44769963011789	11.93262523202601	0.63310108488423
H	2.30932831427915	11.39293023936006	0.24207516234954
H	0.71178925325185	12.02460449473085	-0.17187168151943
H	0.98852931042745	11.33131802598182	1.41878035921584

6: (κ^2-Tp^{iPr2})(OH)(py)Pd

58

Coordinates from ORCA-job TpPdOHPy_PBE0

Pd	5.90154171850913	2.42371878957016	2.67786305798153
O	6.99831408276476	1.19621494094982	3.77074786346739
N	7.37920505303674	3.78069717502695	2.51314755919164
N	7.09529325224187	5.07505281207655	2.78134060395884
N	4.75545222970412	3.71001154165818	1.60513066371566
N	4.74401600562242	5.01133710257748	1.96911514774373
N	3.83741982974720	4.71977627804849	4.62474366884548
N	5.16600135565706	4.91342252787555	4.45628381407133
N	4.38681865851584	1.08176397524660	2.90727320215250
C	8.64989402928668	3.69905609252723	2.12224132375212
C	9.20347508122858	4.98196245116267	2.14923026807698
C	8.19098304350877	5.82739036492545	2.56327669472943
C	9.29408948139366	2.42277672433964	1.71765100084079
C	8.23394326625577	7.29945973561993	2.75346097548445
C	3.98282136277262	3.56892564935814	0.52942272589592
C	3.44789398311026	4.81718821961199	0.19963082195548
C	3.95417678910322	5.70677401392417	1.13106761091943
C	3.78197063981878	2.26478697830357	-0.15304790215673
C	3.71227060063596	7.16738887329015	1.25018502012993
C	3.66218339082111	4.34193766208099	5.88439114050133
C	4.89539066823586	4.30010198820365	6.55283598570422
C	5.83295337840893	4.67919739926181	5.61084360345287
C	2.30637745543211	4.01158006095923	6.40473367451665
C	7.30249490114046	4.82343753061815	5.79467821713794
C	4.61160009718821	-0.22210533248567	2.71929683770505
C	3.61269655145482	-1.16585501160697	2.86572923949119
C	2.34293132925106	-0.74895692689307	3.23172739315399
C	2.11943013010381	0.60256881998747	3.43960967618803
C	3.16441665719634	1.48972263504242	3.26485498782860
B	5.65113573770789	5.51257188954970	3.11311713885391
H	9.87809852478559	1.99577521457227	2.53765305951496
H	9.97677798568173	2.59749117408740	0.88467946084888
H	8.54810613846140	1.68779805715411	1.41580486191705
H	10.21165792779813	5.26042129915052	1.88564644707217
H	9.24049842439875	7.65913753313817	2.54028325705422
H	7.97692960761714	7.58155737167676	3.77684221918930
H	7.53598935763943	7.81046031760568	2.08679688303251
H	3.61150626190128	2.42245807115444	-1.21874402421922
H	2.91171279158019	1.73678332667586	0.24681282919609
H	3.00505248024383	7.47955447600528	0.48182618372659
H	4.63388362613948	7.73974020131967	1.12331775534990
H	1.54331329419949	4.27566397695954	5.67094502946207
H	2.09343875389139	4.54962210071355	7.33184082133981
H	5.08957991349083	4.03174421248751	7.58061784941130
H	2.21242440065946	2.94346326762607	6.62212989590038
H	2.78410942156378	5.04572628985426	-0.61937198040263
H	4.65216831428592	1.61912836012825	-0.02877021247236

H	3.29717019065122	7.42525525750163	2.22688359154176
H	7.83212832967643	1.63974702575027	3.94777701828212
H	1.54026238880172	-1.46665325213573	3.35494711553433
H	3.83771110524742	-2.21070696242217	2.69401412364528
H	5.62289540878259	-0.49869966794458	2.45338608457246
H	3.04003768881575	2.55503442502570	3.41771846668283
H	1.14734506429125	0.97697626721085	3.73424233097744
H	5.62006114092407	6.71551385268453	3.12893583120981
H	7.64744869492382	5.83368463648253	5.56148152245686
H	7.86429480502579	4.12975873298007	5.16460969511775
H	7.55350719866718	4.61424947374642	6.83500186876809

7: $(Tp^{iPr2}Pd)_2(\mu\text{-}OH)_2$

94

Coordinates from ORCA-job Tp2Pd2OH2_PBE0

Pd	0.33466398520884	0.96799766381185	3.59306456693328
O	-1.49255872251779	0.71172310727041	2.74728127299100
N	-0.23022368914982	2.40414512932477	4.86038724126451
N	0.45474073074483	3.56683205426344	4.87969040527045
N	2.20355188693766	1.26244877340379	4.23257275439097
N	2.65270673647891	2.53032933653127	4.36195856529063
N	0.17702320943815	4.31470036994844	2.18222268161329
N	1.36053074917682	3.76029738133395	2.53356339408916
C	-1.23735439615168	2.48846632212312	5.72830457737760
C	-1.20254133974321	3.75395368981629	6.31780734499697
C	-0.11799330005668	4.40772467115088	5.75819503550203
C	-2.18712346133660	1.37316745996058	5.97232838451249
H	-2.50631332233472	1.37255592677913	7.01523158271831
H	-3.08652210044050	1.47645332695585	5.35758664672406
C	0.38026525607989	5.78137748569737	6.02303024381922
H	-0.27740039257230	6.27056763164510	6.74130661153093
H	0.40224649420039	6.38096001491173	5.11057266074996
C	3.15681596381793	0.42938055630291	4.64577681538861
C	4.25806503040977	1.18781747928219	5.04932973128878
C	3.90186024279392	2.51153829980918	4.86315084511836
C	2.99493575306463	-1.04667474995311	4.64690203844735
H	3.61236275290496	-1.49074086788478	5.42812513961317
H	3.31165159227964	-1.47979387677952	3.69337780286804
C	4.69539444908785	3.73540964067031	5.14133098867867
H	4.21098124066179	4.36227891350354	5.89297716883303
H	5.67969377332062	3.44951800306244	5.51146404489385
C	0.14619081353836	4.34339031006571	0.85661158162864
C	1.33399517942483	3.80929329479803	0.33293153936688
C	2.09030828527316	3.45547370258596	1.43384264478224
C	-1.03776422484348	4.87879828667679	0.12830227694972
C	3.45484341796159	2.86159488988540	1.44925857018682
H	4.16632662937239	3.48639527932751	1.99455221949870
H	3.81195900258376	2.76515456015178	0.42354189689592
B	1.73638988616048	3.72966086927126	4.03582448385633
Pd	-1.10786645255552	-1.00704482346177	1.73780276341491

O	0.78797173665470	-0.41489487235970	2.17256508188746
N	-0.61087483834216	-2.69590473157800	0.79649847224429
N	-1.29489189437052	-3.82901362145499	1.06664215737089
C	-0.80631952193172	-4.83334226645458	0.31830688250373
C	0.21918551234623	-4.31907624306787	-0.45615646215814
C	0.30622314402119	-2.96315232742756	-0.13328757283690
C	1.19289546537965	-1.92588094492393	-0.72085140618670
H	0.73450165874105	-1.49258518864133	-1.61472357640201
H	2.14365513517848	-2.36891272257504	-1.01969856138642
C	-1.32154477643676	-6.22504343256911	0.37505042944253
H	-0.72863957894074	-6.85552409338084	-0.28736736146034
H	-1.26260547589012	-6.63365163753811	1.38600850852999
B	-2.48140050140636	-3.82167538456363	2.05441681169086
N	-3.45437646313279	-2.72121149699038	1.58304300397833
N	-3.01519984316928	-1.46019862003615	1.37497696171636
C	-4.01149849174694	-0.74138411862801	0.85901984374315
C	-5.13393046318501	-1.56566778701133	0.74874116217424
C	-4.74340511106215	-2.81136186866381	1.20553646655882
C	-5.54502510045715	-4.05773901985577	1.29768099982671
H	-5.12858221120196	-4.84788986794274	0.66916339741195
H	-6.56335443396044	-3.85444789665327	0.96704358300128
C	-3.85548289132137	0.67386340453034	0.43579120110406
H	-3.53915100256689	0.72598082531793	-0.61016838076620
H	-4.80751868489102	1.19901091767391	0.51951822587287
N	-1.96162952648966	-3.55459370431767	3.48838675354673
N	-0.76157419472348	-4.07656841330388	3.83440286210839
C	-0.61298312911042	-3.86589777788250	5.13550649579324
C	-1.73900408801291	-3.20453180855616	5.65036134779822
C	-2.58379209553640	-3.03039704489917	4.57105002310667
C	-3.93661660288928	-2.41066644409160	4.56705061004127
H	-4.70591645464027	-3.11323789036912	4.23744311758053
H	-4.18500690460539	-2.09500841427486	5.58081543389304
C	0.61744148919652	-4.30664250442937	5.84985054045175
H	0.37765114612869	-4.98458898248359	6.67332851169645
H	1.15715644142306	-3.45729420142334	6.27758052227701
H	-1.87612855188506	4.14082926211848	7.06606269349361
H	-1.72703320005782	0.40810654769560	5.75199168330029
H	1.39234828361715	5.76933152664389	6.43328847463022
H	5.19298872482344	0.81783724244807	5.43948487371400
H	1.95617860506438	-1.33076915847399	4.82529721307293
H	4.82652844768930	4.34206395511331	4.24285491938304
H	1.61448366814712	3.69250371352346	-0.70328680308320
H	3.46747831692273	1.86950327803101	1.90614480133677
H	0.82482084194767	-4.85657568798117	-1.16864882761761
H	1.38243171009374	-1.11969891244875	-0.01293067906577
H	-2.36561861305929	-6.27875593521133	0.05893175168764
H	-6.10735427436136	-1.28974551616460	0.37512458021328
H	-5.58346123703257	-4.43818594349084	2.32061075055311
H	-3.10883655245849	1.18708178085942	1.04102622016199
H	-1.92362131012493	-2.89166477559910	6.66725871314874

H	-3.98914617022907	-1.53386374711950	3.91737118879578
H	1.28895360967700	-4.82523374193243	5.16387529306575
H	-0.75710043129629	5.69404282039198	-0.54379887044162
H	-1.77810592947154	5.25686314220309	0.83492641533197
H	-1.51292396467616	4.10628811661559	-0.48300845653173
H	-2.18727907575457	0.55483993045354	3.39494567843141
H	1.29210543809104	-1.15429031458483	2.52714404711255
H	-3.04691914062231	-4.88057826814406	1.97901033996440
H	2.29875169668881	4.73695075363661	4.37671139170251

8: Pd_2Cl_6

Coordinates from ORCA-job Pd2Cl6

Cl	1.74897620659226	4.44633038026784	11.99063560337067
Cl	2.53571069213309	5.98845511998447	9.17010589799163
Cl	0.99004010512611	3.62458882541883	7.50102311936401
Pd	1.39627582628891	4.08034783768505	9.70353357539037
Cl	0.25544952182355	2.14666256711681	10.37236459640115
Cl	-0.53131416326751	0.60453640686705	13.19280350191436
Cl	1.00954588921105	2.97128137883946	14.86224188173813
Pd	0.60689592209252	2.51339748382046	12.65952182382961

References:

(1) Johnson III, R. D. NIST Standard Reference Database Number 101. In *NIST Computational Chemistry Comparison and Benchmark Database*; 2022.

(2) Krause, M. O.; Oliver, J. H. Natural Widths of Atomic K and L Levels, Kα X-ray Lines and Several KLL Auger Lines. *J. Phys. Chem. Ref. Data* **1979**, *8* (2), 329–338.

(3) Genc, A.; Marlowe, J.; Jalil, A.; Belzberg, D.; Kovarik, L.; Christopher, P. A Versatile Machine Learning Workflow for High-Throughput Analysis of Supported Metal Catalyst Particles. *Ultramicroscopy* **2025**, *271*, 114116.

(4) Lassalle-Kaiser, B.; Boron, T. T. I.; Krewald, V.; Kern, J.; Beckwith, M. A.; Delgado-Jaime, M. U.; Schroeder, H.; Alonso-Mori, R.; Nordlund, D.; Weng, T.-C.; Sokaras, D.; Neese, F.; Bergmann, U.; Yachandra, V. K.; DeBeer, S.; Pecoraro, V. L.; Yano, J. Experimental and Computational X-Ray Emission Spectroscopy as a Direct Probe of Protonation States in Oxo-Bridged Mn^{IV} Dimers Relevant to Redox-Active Metalloproteins. *Inorg. Chem.* **2013**, *52* (22), 12915–12922.
